# Identification of a polyamine-related signature and six novel prognostic biomarkers in oral squamous cell carcinoma

**DOI:** 10.3389/fmolb.2023.1073770

**Published:** 2023-01-17

**Authors:** Jiezhang Tang, Xuechen Wu, Bo Cheng, Yajie Lu

**Affiliations:** ^1^ Department of Plastic and Reconstructive Surgery, Xijing Hospital, Fourth Military Medical University, Xi’an, China; ^2^ Department of Stomatology, Zhongnan Hospital of Wuhan University, Wuhan, China; ^3^ Department of Clinical Oncology, Xijing Hospital, Fourth Military Medical University, Xi’an, China

**Keywords:** oral squamous cell carcinoma, bioinformatics analyses, The Cancer Genome Atlas, polyamines, prognostic model, immune infiltration

## Abstract

Elevated polyamine levels are required for tumor transformation and development; however, expression patterns of polyamines and their diagnostic potential have not been investigated in oral squamous cell carcinoma (OSCC), and its impact on prognosis has yet to be determined. A total of 440 OSCC samples and clinical data were obtained from The Cancer Genome Atlas (TCGA) and Gene Expression Omnibus (GEO). Consensus clustering was conducted to classify OSCC patients into two subgroups based on the expression of the 17 polyamine regulators. Polyamine-related differentially expressed genes (PARDEGs) among distinct polyamine clusters were determined. To create a prognostic model, PARDEGs were examined in the training cohorts using univariate-Lasso-multivariate Cox regression analyses. Six prognostic genes, namely, “*CKS2*,” “*RIMS3*,” “*TRAC*,” “*FMOD*,” *CALML5*,” and “*SPINK7*,” were identified and applied to develop a predictive model for OSCC. According to the median risk score, the patients were split into high-risk and low-risk groups. The predictive performance of the six gene models was proven by the ROC curve analysis of the training and validation cohorts. Kaplan–Meier curves revealed that the high-risk group had poorer prognosis. Furthermore, the low-risk group was more susceptible to four chemotherapy drugs according to the IC50 of the samples computed by the “pRRophetic” package. The correlation between the risk scores and the proportion of immune cells was calculated. Meanwhile, the tumor mutational burden (TMB) value of the high-risk group was higher. Real-time quantitative polymerase chain reaction was applied to verify the genes constructing the model. The possible connections of the six genes with various immune cell infiltration and therapeutic markers were anticipated. In conclusion, we identified a polyamine-related prognostic signature, and six novel biomarkers in OSCC, which may provide insights to identify new treatment targets for OSCC.

## 1 Introduction

The three primary polyamines, namely, putrescine, spermidine, and spermine (McNamara et al., 2021), are plentiful both inside the cell and outside it. Polyamines are multifunctional polycations which are necessary for practically all living things (Novita Sari et al., 2021). Polyamines, because of their cationic nature, could interact with macromolecules such as DNA, RNA, phospholipids, and proteins to increase gene regulation *via* epigenetic and chromatin structural change, mRNA structure stabilization, cell growth, proliferation and differentiation, and ion channel regulation (Pegg, 2009; Murray-Stewart et al., 2018). It is used by cells throughout the body to support growth, and microorganisms can interact with polyamines to reduce the expression of pro-inflammatory genes (Holbert et al., 2022). The downstream of numerous significant carcinogenic pathways is the polyamine metabolism (Casero et al., 2018). The polyamine pathway is a viable target for anti-cancer treatment since it is frequently dysregulated in cancer. Additionally, when polyamine production is downregulated by pharmacological and genetic techniques, it will cause cancer cells to senescence and undergo apoptosis (Minois et al., 2011). It may be argued that polyamine levels could serve as a cancer marker because they promote proliferation as well (McNamara et al., 2021), and the increased demand of tumor cells for polyamines, to some extent, shows that targeted polyamines are important strategies in cancer treatments. Polyamines and polyamine metabolites measured under humoral environment have shown potential as biomarkers for different cancers (Liu et al., 2018; Nakajima et al., 2018). At present, polyamine inhibitors and analogs are effective for cancer in experimental animal models and in clinic; for example, α-difluoromethylornithine (DFMO) was evaluated as a potential combined cancer therapy drug (Holbert et al., 2022). For another example, spermidine and spermine that have been reported could stimulate apoptosis of macrophages stimulated by *Helicobacter pylori* (Chaturvedi et al., 2004), and DFMO could inhibit the apoptosis (Gobert et al., 2002). In various malignancies, the involvement of polyamines in the development of cancer has been demonstrated. Colorectal cancer (Snezhkina et al., 2016) is regulated by the oncogenic transcription factors c-MYC and C/EBP β *via* a number of important enzymes in the polyamine metabolic pathway. Ornithine decarboxylase (ODC) has been identified as an effective carcinogenic transformation factor, which is the most effective transcriptional target in neuroblastoma (Lu et al., 2003). Polyamines are abundant in breast tissue and can promote cell proliferation by promoting growth factor receptors (Çelik et al., 2017). In prostate cancer, the concentration of spermine is related to the malignant grade of cancer (Tsoi et al., 2016), and it becomes a biomarker of the malignant degree of cancer.

At present, studies on polyamines and oral cancer focus on the detection of the saliva metabolism. Japanese scholars have characterized the metabolic changes in patients' saliva samples (Ohshima et al., 2017) among the potential 25 metabolites, and the polyamine metabolism showed significant changes. In the study of Ishikawa et al. (2019), salivary ornithine can be utilized to distinguish between oral epithelial dysplasia and oral squamous cell cancer as an intermediate metabolite in the urea cycle, which is a well-known metabolic marker of various tumors (Nijakowski et al., 2022). These recently discovered polyamine-related genes may aid in the development of an improved mechanistic comprehension of OSCC, which may provide a productive insight to the clinical application of the condition.

**FIGURE 1:**
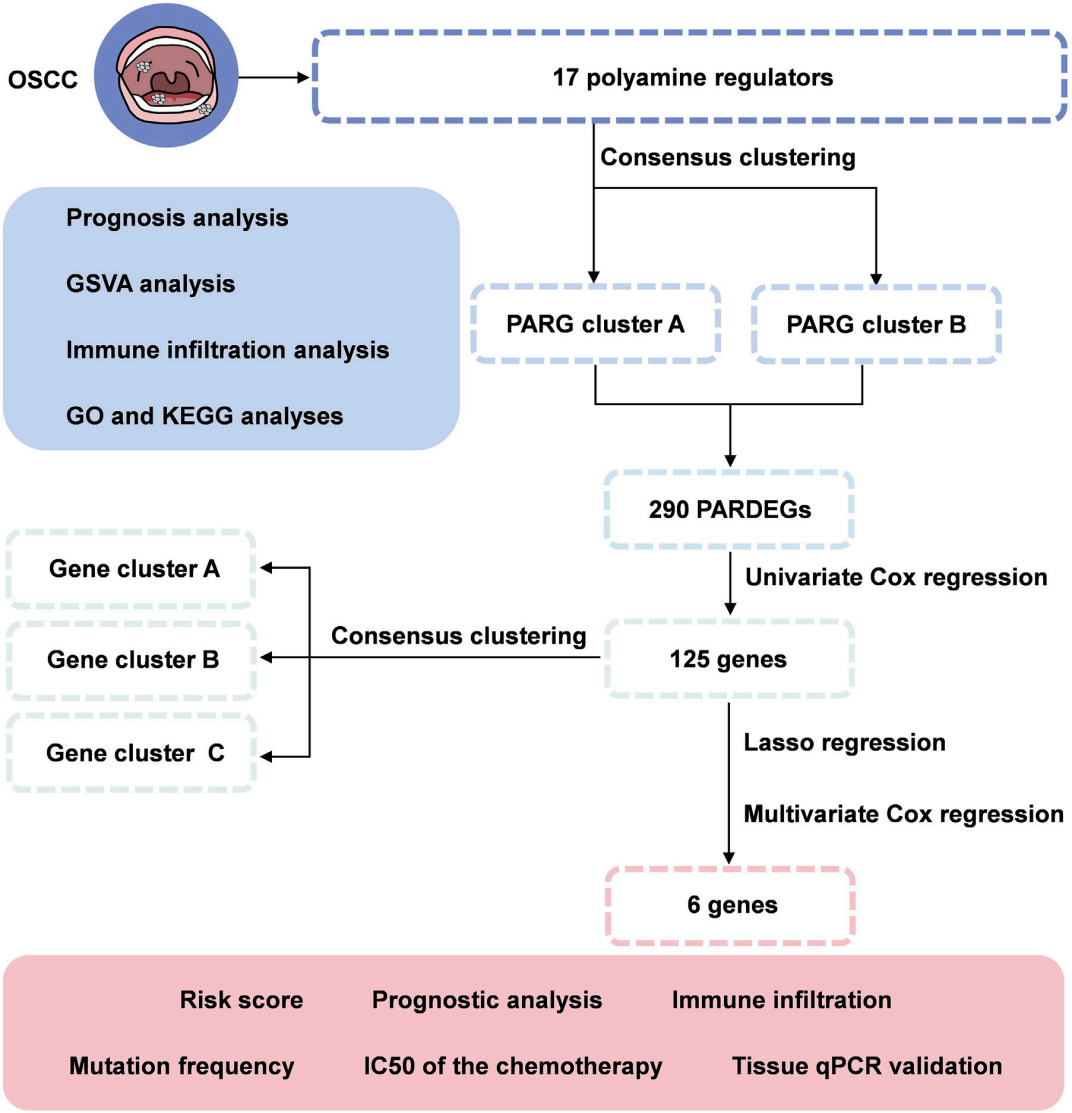
Flowchart of identifying a polyamine-related signature and six novel prognostic biomarkers in oral squamous cell carcinoma.

In our study, we combined genomic data from 420 OSCC samples from TCGA and GEO datasets. We discovered two distinct polyamine modification patterns and found that the immune cell infiltration is different in clusters, indicating that the modification played a vital role in shaping individual tumor microenvironment characterizations. Furthermore, we developed a scoring model to assess individual tumor polyamine alteration patterns and predict patients' clinical response to chemical therapy (Figure 1). These findings showed that polyamine alteration is critical in establishing various tumor immune microenvironment profiles and driving therapeutic intervention approaches for oral squamous cell carcinoma.

## 2 Materials and methods

### 2.1 Data collection

We obtained the sequence data for 323 samples with OSCC from TCGA and 97 samples from the NCBI GEO database (GSE41613). A total of 17 polyamine signatures were extracted from Holbert’s research (Holbert et al., 2022) and four gene sets in the Gene Set Enrichment Analysis database as follow: GOBP_REGULATION_OF_POLYAMINE_TRANSMEMBRANE_TRANSPORT, GOBP_PUTRESCINE_METABOLIC_PROCESS, GOBP_PUTRESCINE_BIOSYNTHETIC_PROCESS, and GOBP_POLYAMINE_TRANSMEMBRANE_TRANSPORT.

### 2.2 Consensus clustering analysis of 17 polyamine regulators

We chose to investigate potential molecular subgroups by using the “ConsensusClusterPlus” R package (Wilkerson and Hayes, 2010) to perform consensus clustering analysis to identify distinct polyamine modification patterns based on the expression of 17 polyamine regulators (*AGMAT*, *AMD1*, *ATP13A2*, *AZIN1*, *AZIN2*, *OAZ1*, *OAZ2*, *OAZ3*, *ODC1*, *AOC1*, *PAOX*, *SAT1*, *SAT2*, *SMOX*, *ARG1*, *SRM*, and *SMS*).

### 2.3 Functional analyses

To identify differently expressed genes in distinct groups, the “limma” package in R was used. The additional verification of the GO processes in BP, CC, MF, and KEGG pathways was associated with signature analysis for the differentially expressed genes in the cluster; the “clusterProfiler” package in R (Yu et al., 2012) was applied in each sample with a statistical threshold of *p* < 0.05.

### 2.4 Tumor mutational burden and immune cell infiltration

Patients' single-nucleotide variant data were used to estimate the tumor mutational burden (TMB). The single sample gene set enrichment analysis (ssGSEA) algorithms assessed the 28 immune cells infiltration in the OSCC microenvironment.

### 2.5 Differentially expressed genes identified between unique polyamine modification patterns

Our previous consensus clustering algorithm classified patients into two distinct polyamine modification patterns, and polyamine-related differentially expressed genes (PARDEGs) among distinct polyamine phenotypes were determined. R package was applied to assess PARDEGs in OSCC samples among different clusters. Gene expression data were normalized to calculate the differentially expressed statistics. The significance filtering criteria of PARDEGs were set as an adjusted value of *p* < 0.05.

### 2.6 Polyamine-related risk signature construction and validation

In order to create an optimal polyamine-related risk signature based on linear integration of the regression coefficient obtained and the expression level of the chosen genes, we generated a polyamine-related score pattern using LASSO regression with the “glmnet” R package using intersecting genes from the public datasets. The risk score was computed as follows: risk score=(0.2340×CKS2)+(−0.1522×RIMS3)+(−0.1624×TRAC)+(−0.1918×FMOD)+(−0.0871×CALML5)+(−0.0934×SPINK7 ).

### 2.7 Survival analysis

To compare the OS of different clusters of OSCC patients, the Kaplan–Meier analysis and the “survminer” programs were utilized. The “survivalROC” test was used to create ROC curves in order to assess the accuracy of the risk signatures in predicting the outcomes of OSCC patients. The bigger the AUC, the better the risk model’s prediction power is.

### 2.8 Quantitative real-time PCR

Total RNA extraction using TRIzol reagent (Servicebio, Wuhan, China) was performed as previously described (Wu et al., 2022). Total RNA was then converted to a first-strand cDNA by ReverTra Ace (Toyobo, Tokyo, Japan) and subjected to qRT-PCR (Bio-Rad CFX96, America) with ChamQ Universal SYBR qPCR Master Mix (Vazyme Biotech, Nanjing, China). In total, three duplicates of each sample were analyzed. Once the expression levels of GAPDH were analyzed, the results were calculated in 2^-ΔΔCt. The target genes' primer sequences are included in Supplementary Table S3.

### 2.9 Drug sensitivity and interactions

Furthermore, we used the R package “pRRophetic” (Geeleher et al., 2014) to assess each OSCC patient’s therapy response, which is based on the 50% inhibitory concentration (IC50) acquired from the Genomics of Drug Sensitivity in Cancer (GDSC) website.

### 2.10 Statistical analysis

The data were analyzed by R software (https://www.r-project.org/). R packages (ESTIMATE, glmnet, ggplot2, GSVA, limma, survminer, and survival) were applied for data analysis and graph plotting. The median value of tumor purity or risk scores was treated as the cutoff value for the two subgroups. The qPCR results were analyzed by GraphPad (version 9.0.0). In order to compare the statistical differences between the two groups, the Student’s *t*-test was employed. When there were more than two groups, Kruskal–Wallis and one-way ANOVA tests were used. The *p*-value was always two-sided; a value of *p* < 0.05 was considered statistically different (*, *p* < 0.05; **, *p* < 0.01; ***, *p* < 0.001).

## 3 Results

### 3.1 Outline of polyamine-related gene expression changes in OSCC and a PPI network

**FIGURE 2:**
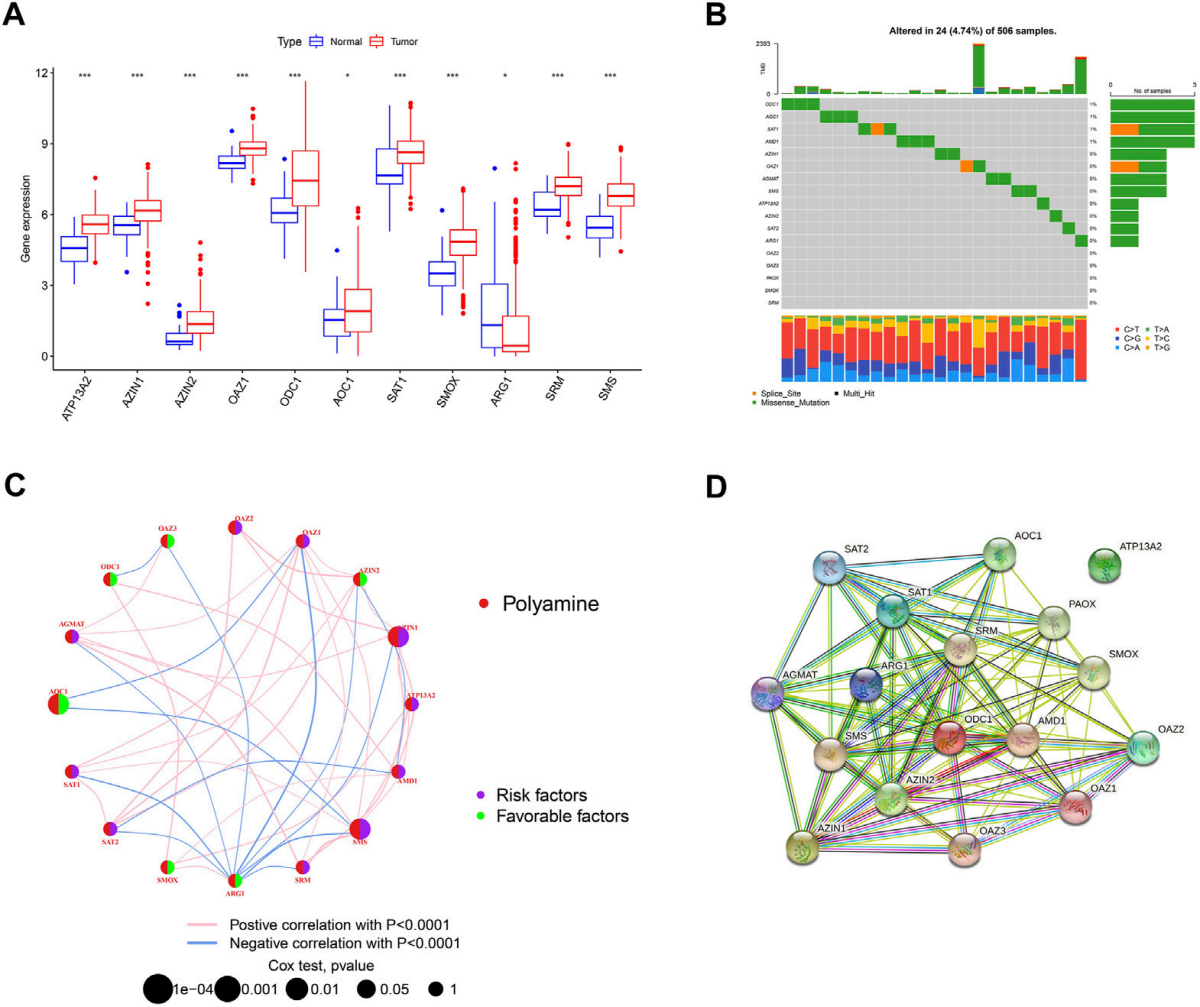
Overview of genetic and prognostic information of polyamine regulators in OSCC. **(A)** Boxplot of 17 polyamine regulators expression in OSCC and its adjacent normal tissue. **(B)** Waterfall plot of the polyamine regulators altered in OSCC samples. **(C)** Interaction of the polyamine regulators. Size of each cell represents the survival effect of each gene. Red represents a positive correlation, whereas blue indicates a negative correlation. **(D)** PPI network map showed the interaction of the 17 polyamine regulators.

We first investigated the expression of 17 polyamine regulators (*AGMAT*, *AMD1*, *ATP13A2*, *AZIN1*, *AZIN2*, *OAZ1*, *OAZ2*, *OAZ3*, *ODC1*, *AOC1*, *PAOX*, *SAT1*, *SAT2*, *SMOX*, *ARG1*, *SRM*, and *SMS*) in OSCC patients. The results showed that most polyamine regulators were expressed higher in neoplastic tissues in OSCC patients than in their normal tissues. However, ARG1 had low expression in cancer tissues and high expression in normal tissues (Figure 2A). The mutation analysis of OSCC patients showed that only 4.74% of 506 samples had polyamine regulator gene alterations. The mutations frequency was concentrated in *ODC1*, *AOC1*, *SAT1*, and *AMD1*, most of which were missense mutations (Figure 2B).

We made a correlation analysis on these polyamine genes to see whether they interact with each other. The regulator network depicted the complete picture of the interactions of the 17 polyamine regulators, the regulator interconnections, and their prognostic value in OSCC patients. Results in Figure 1C show that 6/17 of the polyamine regulators are favorable factors, and among them, ARG1 has the most abundant blue lines (representing negative correlation), which shows its regulatory importance in tumors. In addition, AMD1, AZ1N1, ARG1, and OAZ1 have the most related lines with other genes, indicating that cross-talk among the regulators plays a role in regulation (Figure 2C). A PPI (protein–protein interaction) network shows the interaction between proteins encoded by polyamine genes. ATP13A2 has no interaction with other proteins but has connections with other genes in the correlation network, suggesting that there may still be proteins encoded by intermediate genes that have not been included in tumor development. For others, pink lines between *SRM* and *ODC1*, *AMD1* and *AZIN2*, *etc*., indicated that there has been experimental verification of interaction (experimentally determined). Most of the gene connections of polyamines are still emerald green (text mining), which means they have research potential and need to be further explored (Figure 2D).

### 3.2 Polyamine modification patterns characterized by gene expression

**FIGURE 3:**
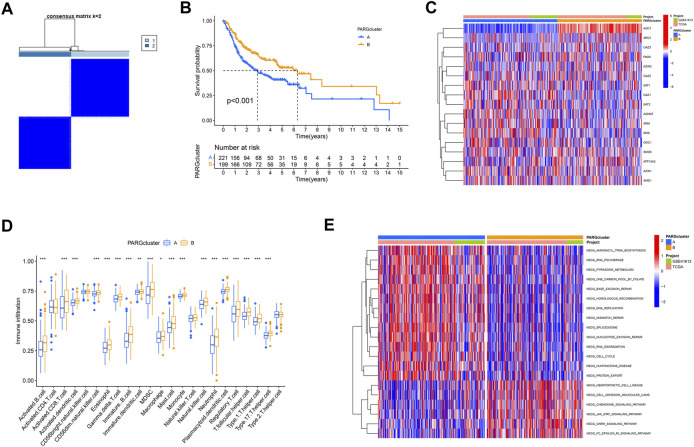
Construction of two polyamine-related gene (PARG) clusters in OSCC patients. **(A)** Consensus clustering of OSCC patients. **(B)** OS curves of OSCC patients in two PARG clusters. **(C)** Heatmap of 17 polyamine regulator expression in two clusters. **(D)** Quantity of immunological infiltration cells as determined by ssGSEA between PARG clusters. **(E)** Heatmap of GSVA in the KEGG pathway between PARG clusters.

Based on the hypotheses of polyamine regulators with OSCC progression, we identified two distinct polyamine-related gene (PARG) clusters (Figure 3A; Supplementary Figures S1A–J); thus, we divided all patients into cluster PARG-A (n = 221) and PARG-B (n = 199) by consensus clustering. PARG-A exhibited the worst prognosis, whereas PARG-B had a prominent survival advantage (Figure 3B).

After the differential analysis of all polyamine-related genes (PARGs), heatmap showed that *AOC1* showed low expression in PARG-B with poor prognosis, and its high expression in PARG-A was helpful to the prognosis of OSCCs. Also, although the expression of an *ARG1* gene is lower than that of other PARGs in OSCC patients (Figure 2A), it can still be seen that its high expression indicates a better prognosis (Figure 3C).

### 3.3 Immune cell infiltration and pathways in two PARG clusters

We also analyzed the differences between the two clusters of immune infiltrating cells. We used ssGSEA to create a boxplot to visualize and compare the 23 immune infiltrating cell subtypes among distinct clusters (Figure 3D). Compared to PARG-A, the expression of most lymphocytes and myeloid cells in PARG-B was higher, such as activated CD8^+^ T cells, T follicular helper cell, and Type 1/17 T helper cells. Natural killer cells (NK cells), which are considered to be the next leading role of cellular immunotherapy (Laskowski et al., 2022), have higher infiltration in PARG-B with better prognosis.

Moreover, we used GSVA to explore the biological molecular changes of PARG-A and B. As shown in the result in the heatmap (Figure 3E), PARG-A has a poor prognosis, concentrated in metabolic-related pathways, such as pyrimidine metabolism, one carbon pool by folate, aminoacyl-tRNA biosynthesis, and RNA polymerase. PARG-B is concentrated in a chemokine signaling pathway. Chemokines are small molecular weight cytokines, whose main role is to recruit leukocyte subsets under stable and pathological conditions, that transmit cell signals after binding with the chemokine receptor, which are expressed on the cell surface. Cell adhesion molecule cam has the function of maintaining the normal tissue structure, regulating immune response and inflammatory response, and is also highly expressed in PARG-B.

Based on our findings, the two polyamine modification patterns had distinct immune infiltration characteristics.

### 3.4 GO term and the KEGG pathway analyses

Despite the fact that the consensus clustering technique based on PARG expression categorized OSCC patients into two polyamine modification phenotypes, the underlying genetic modifications and expression perturbations within these phenotypes remained unknown. Based on these questions, we investigated the possible polyamine-related transcriptional expression changes in OSCC across two polyamine modification types. We identified 289 polyamine-related differentially expressed genes (PARDEGs) (Supplementary Table S1) between the two PARG clusters.

**FIGURE 4:**
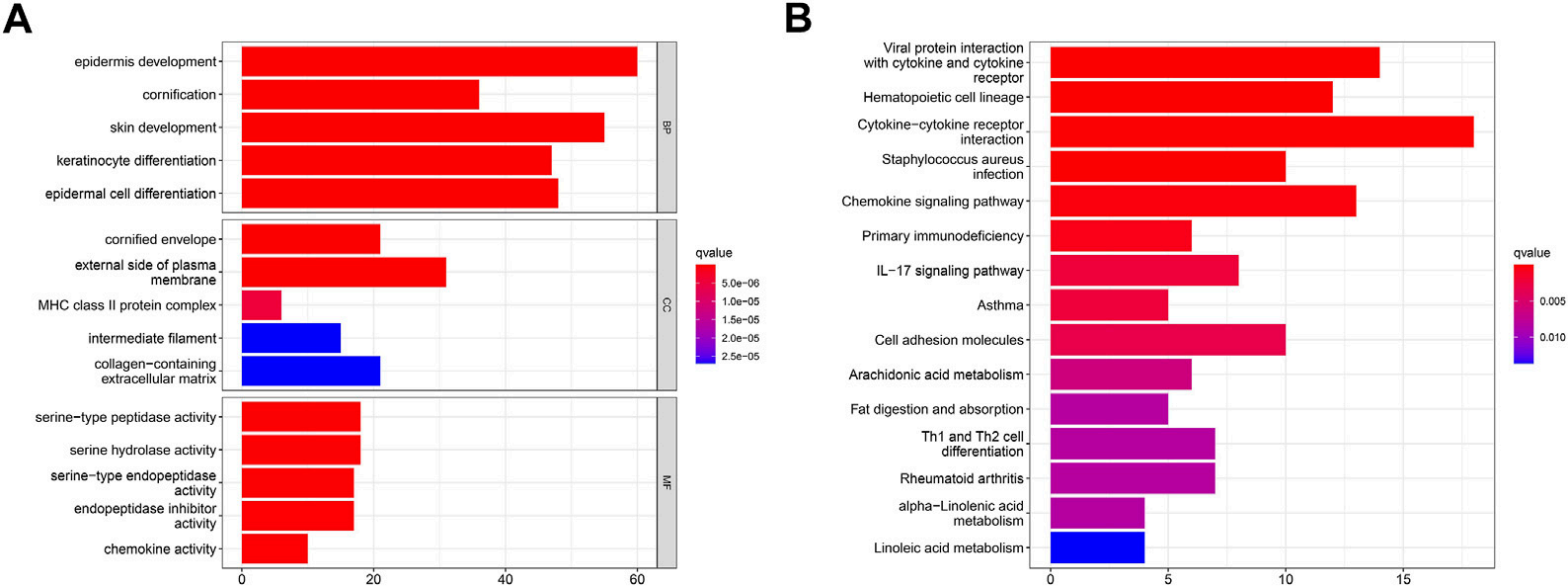
Functional annotation of 17 polyamine regulators using GO terms and the KEGG pathway. GO **(A)** and KEGG **(B)** analyses of the identified genes.

The Gene Ontology (GO) and Kyoto Encyclopedia of Genes and Genomes (KEGG) analyses of PARDEGs were carried out. The biological process (BP) (Figure 4A) of PARDEGs was concentrated in the occurrence and development of epithelial development. At the same time, it was concentrated in the human immune response, acute human response, and other immune-related pathways. The most abundant cellular component (CC) influenced the immune microenvironment, in which the external side of plasma is more expressed. The serine-type peptidase activity and serine hydrolase activity were the two most prevalent molecular function (MF) terms. Also, other pathways in the KEGG pathway analysis showed that the cytotoxic receptor and chemokine signaling pathway were the most abundant pathways (Figure 4B).

### 3.5 PARDEGs construct phenotypes in OSCC

**FIGURE 5:**
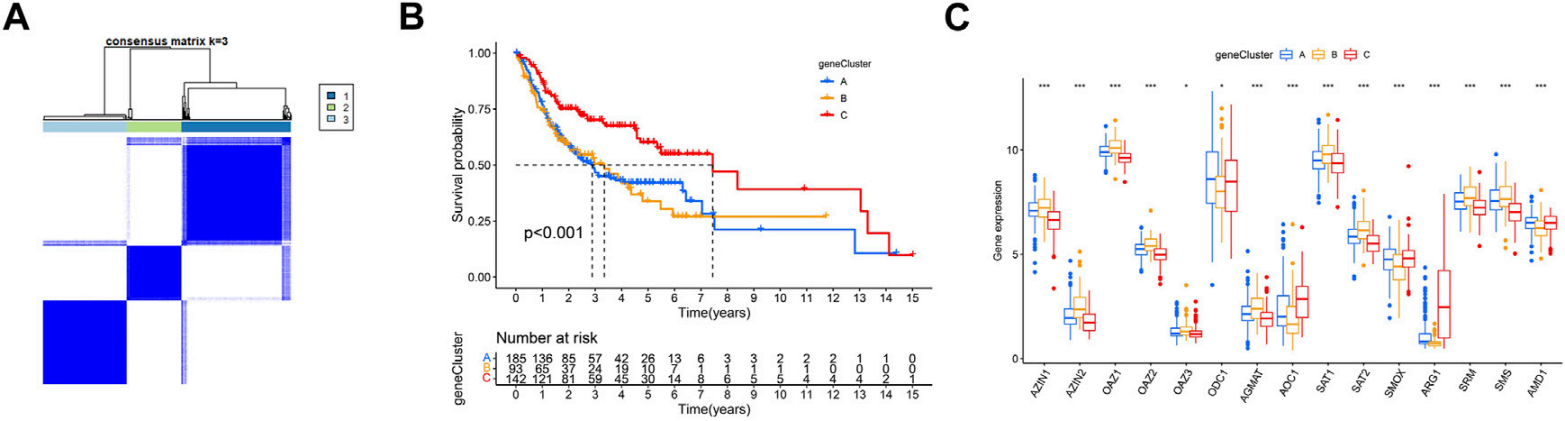
Identification of polyamine-related differentially expressed gene (PARDEG) clusters. **(A)** Consensus clustering of OSCC patients based on PARDEGs. **(B)** Comparison of the three cluster survival probability of OSCC patients. **(C)** Polyamine marker expression in three clusters.

We first conducted univariate Cox regression among the 289 PARDEGs, and we got 125 prognostic genes in total, as shown in Supplementary Table S2. Then, we performed consensus clustering analysis based on the 125 prognostic genes and obtained three stable transcriptomic phenotypes (Figure 5A; Supplementary Figures S2A–J).

Among them, the survival rate of group B (n = 93) is the worst, while that of group C (n = 142) is more advantageous in the long survival probability (Figure 5B). By analyzing the polyamine regulator expression in A, B, and C groups, we found that the previously mentioned favorable factors *ARG1* and *AOC1* were highly expressed in the C group; in other words, they were expressed low in A and B groups (Figure 5C).

**FIGURE 6:**
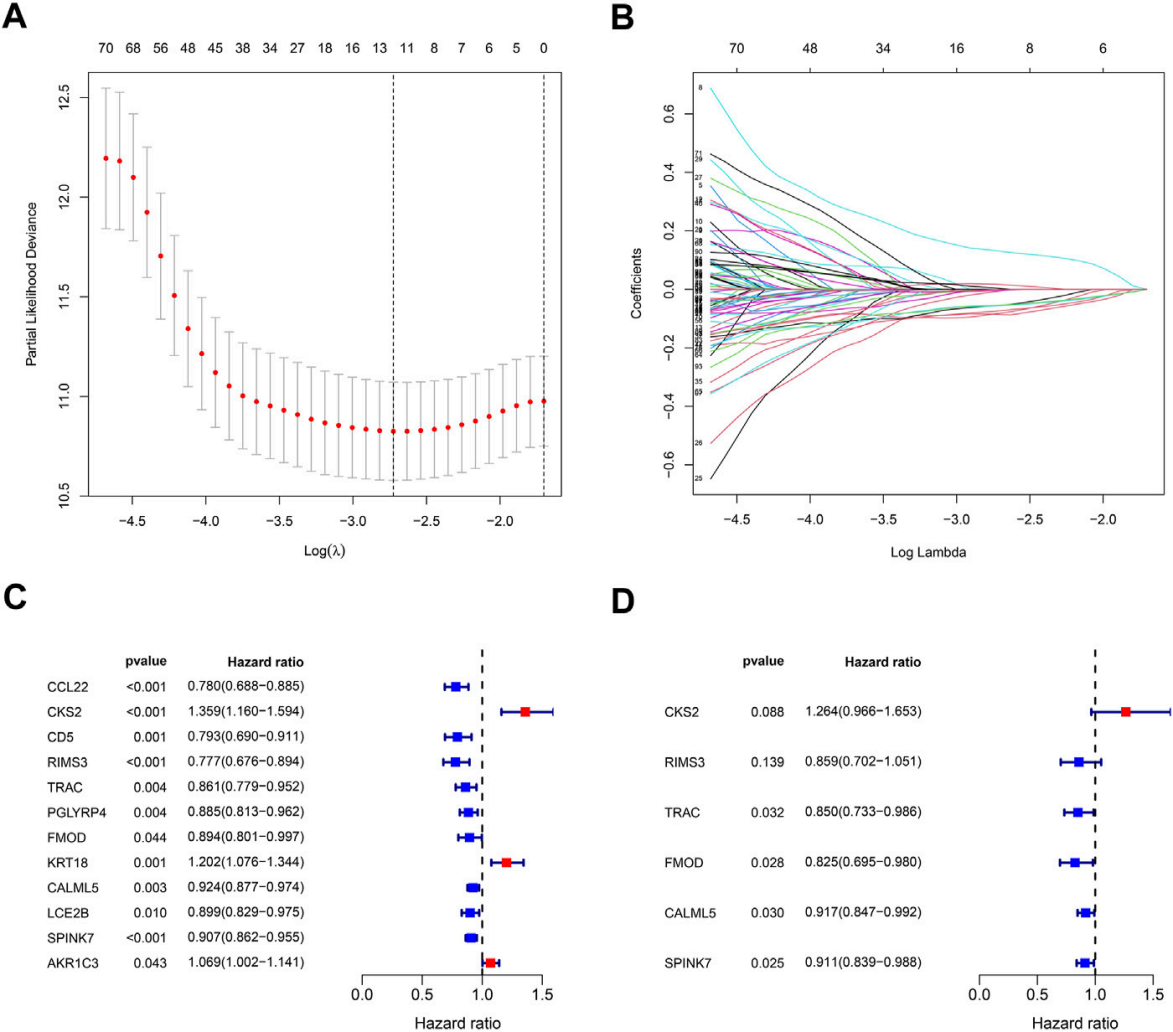
Stepwise identification of PARG risk signature of the model. **(A)** Cross-validation for tuning parameter selection in the proportional hazards model. **(B)** LASSO coefficient of the PARDEGs. **(C)** Forest plot of the univariate Cox regression analysis in DEGs. **(D)** Forest plot of the multivariate Cox regression analysis in DEGs.

In order to build a prognostic model related to the oral squamous cell carcinoma, the LASSO regression for these genes was carried out (Figures 6A, B), and biomarkers of the 12 genes were screened. Additionally, by the univariate Cox regression, we can identify whether these genes are risk or preventive factors (Figure 6C). Finally, multivariate Cox regression was used to select the final six penalty genes (Figure 6D). At this time, the risk of overfitting is minimizing.

### 3.6 The development of the risk score by PARDEGs and prognostic analysis

**FIGURE 7:**
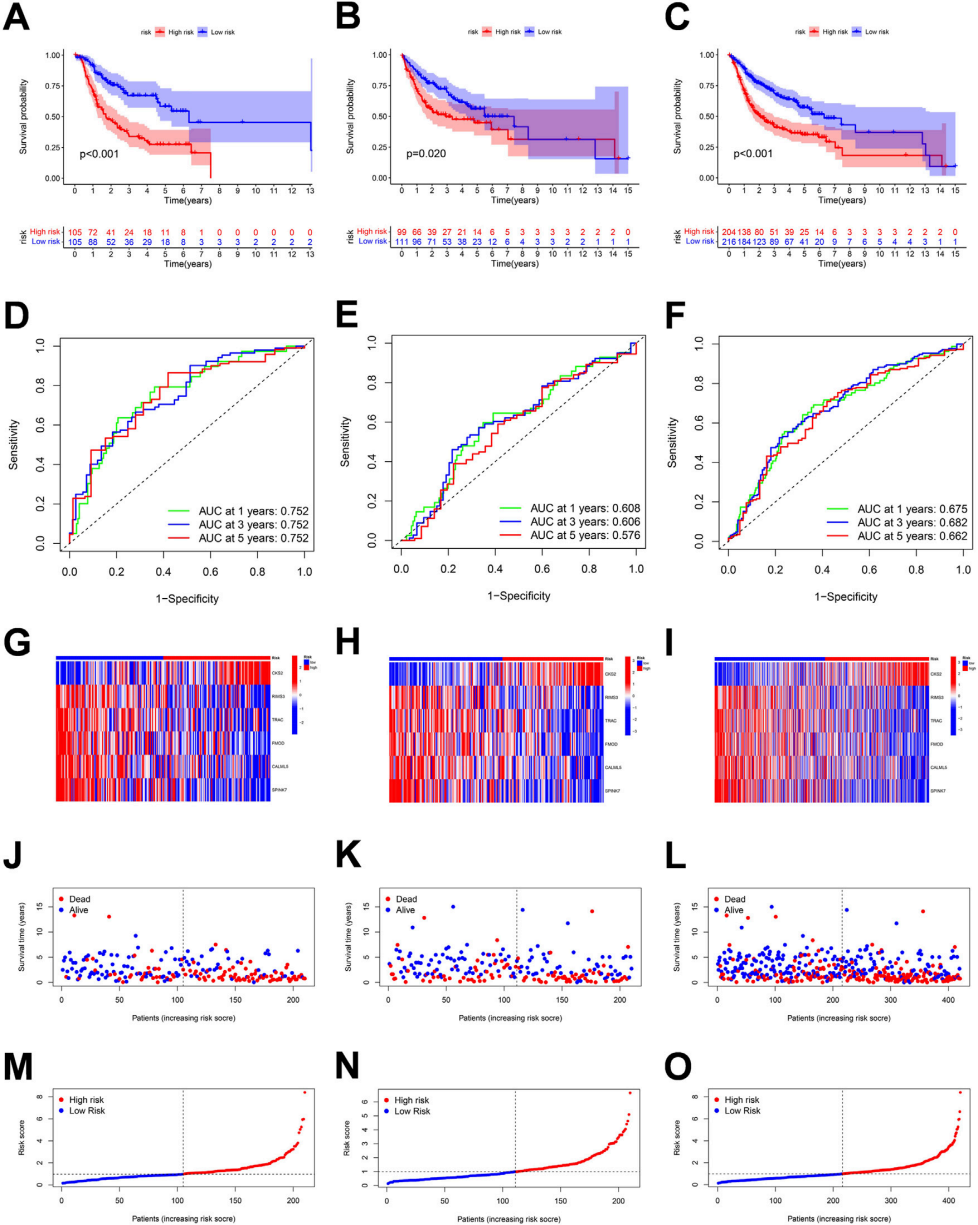
Correlation between the risk score and overall survival of OSCC patients in the training, validation, and the whole cohort. **(A**–**C)** Overall survival (OS) of the high-risk group was significantly shorter than that of the low-risk group. **(D**–**F)** ROC curve and the areas under the curve for predicting a 1-, 3-, and 5-year OS in OSCC patient. **(G**–**I)** Heatmap of six genes’ expression in the training, test, and total cohorts. **(J**–**O)** Based on the polyamine-related risk score, groups are distributed. Scatterplot showed the variations in OSCC patients' survival rates between high-risk and low-risk categories.

According to the six penalty genes, namely, “*CKS2*,” “*RIMS3*,” “*TRAC*,” “*FMOD*,” “*CALML5*,” and “*SPINK7*,” the model was established and its effectiveness was verified. The risk scores were calculated as the following formula: $risk\;score=\sum_{i=1}^{n}{expr}_{genei}\times {coefficient}_{genei}$ . Patients were randomly divided into training and validation cohorts. In our study, the survival curves (Figures 7A–C) of the training cohort (*p* < 0.001), the validation cohort (*p* = 0.02), and the whole cohort (*p* < 0.001) could better distinguish the survival of high-risk groups, while the ROC working curve was also highly efficient for a 1-, 3-, and 5-year overall survival (OS) that were 0.752, 0.752, and 0.752 and 0.608, 0.606, and 0.576 in the training and validation cohorts, respectively (Figures 7D–F). It was found that *CKS2* was a risk gene for OSCC, and its high expression was associated with poor prognosis (Figures 7G–I). We replaced all patients with risk points, and the model can more effectively identify between patients with OSCC of high-risk (red points) and low-risk (blue points) points (Figures 7J–O **)**. The median value served as the threshold to distinguish between high- and low-risk groups. As shown in Figures 7J–L, the number of patient deaths increased significantly as the risk score increased. Figures 7M–O show the distributions of risk scores among patients.

### 3.7 Correlation between PARGs and its related clusters with risk scores

**FIGURE 8:**
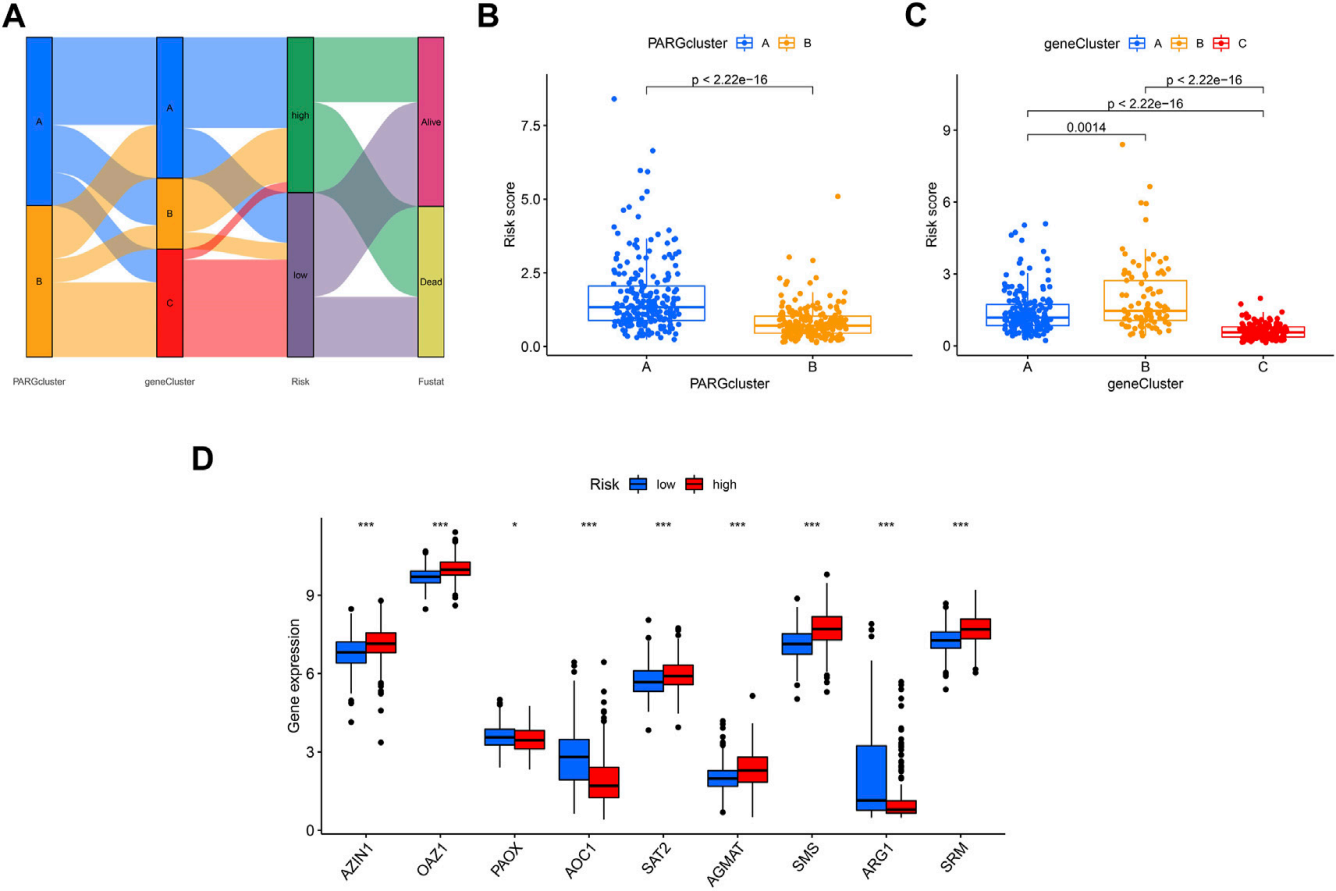
Comparison of the effectiveness of risk scores in different groups. **(A)** Sankey plot shows the OSCC patients’ distribution in different states of our analyses. **(B)** Risk score in A and B clusters related to PARGs. **(C)** Risk score in A, B, and C clusters divided by the PARDEG cluster-related genes. **(D)** High- and low-risk groups expressed PARGs significantly different.

The Sankey plot shows the flow of the OSCC patients’ survival and death status from the first clustering analysis to the second clustering analysis and then to the final risk-related group (Figure 8A). The risk scores of the two groups were consistent with the previous survival prognosis (Figures 8B, C). Again, we examined the expression of genes related to polyamines in the two groups determined by the risk score; as shown in Figure 8D, *PAOX*, *AOC1*, and *ARG1* were expressed higher in the low-risk group, while *AZIN1*, *OAZ1*, *SAT2*, *AGMAT*, *SMS*, and *SRM* were the opposite.

### 3.8 Correlation between immune infiltration cells and risk scores

**FIGURE 9:**
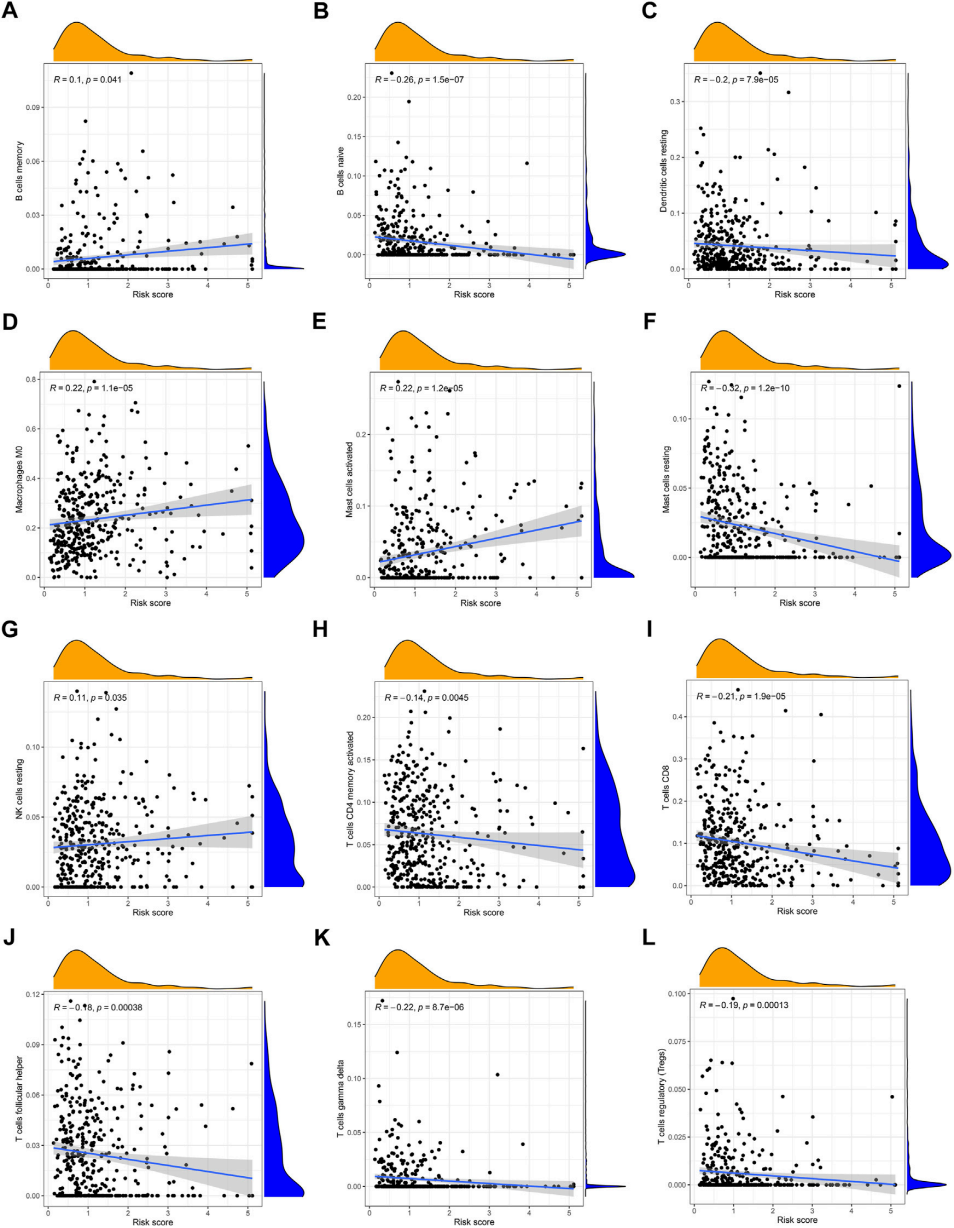
Correlation between the risk score and the immune cell infiltration by ssGSEA **(A)** memory B cells, **(B)** naive B cells, **(C)** resting dendritic cells, **(D)** M0 macrophages, **(E)** activated mast cells, **(F)** resting mast cells, **(G)** resting NK cells, **(H)** activated memory CD4^+^ T cells, **(I)** CD8^+^ T cells, **(J)** follicular helper T cells, **(K)** gamma delta T cells, and **(L)** regulatory T cells.

Simultaneously, we examined the expression of 22 immune cell infiltration correlated with risk scores by ssGSEA. There was a significant inverse correlation between the risk score and the naïve B cells, resting mast cells, resting dendritic cells, activated memory CD 4 + T cells, CD8^+^ T cells, helper follicular T cells, gamma delta T cells, and regulatory T cells. The rest cell types were positively correlated with risk scores such as memory B cells, M0 macrophages, activated mast cells, and resting NK cells (Figures 9A–L).

### 3.9 Differences in mutation frequency between high- and low-risk groups

**FIGURE 10:**
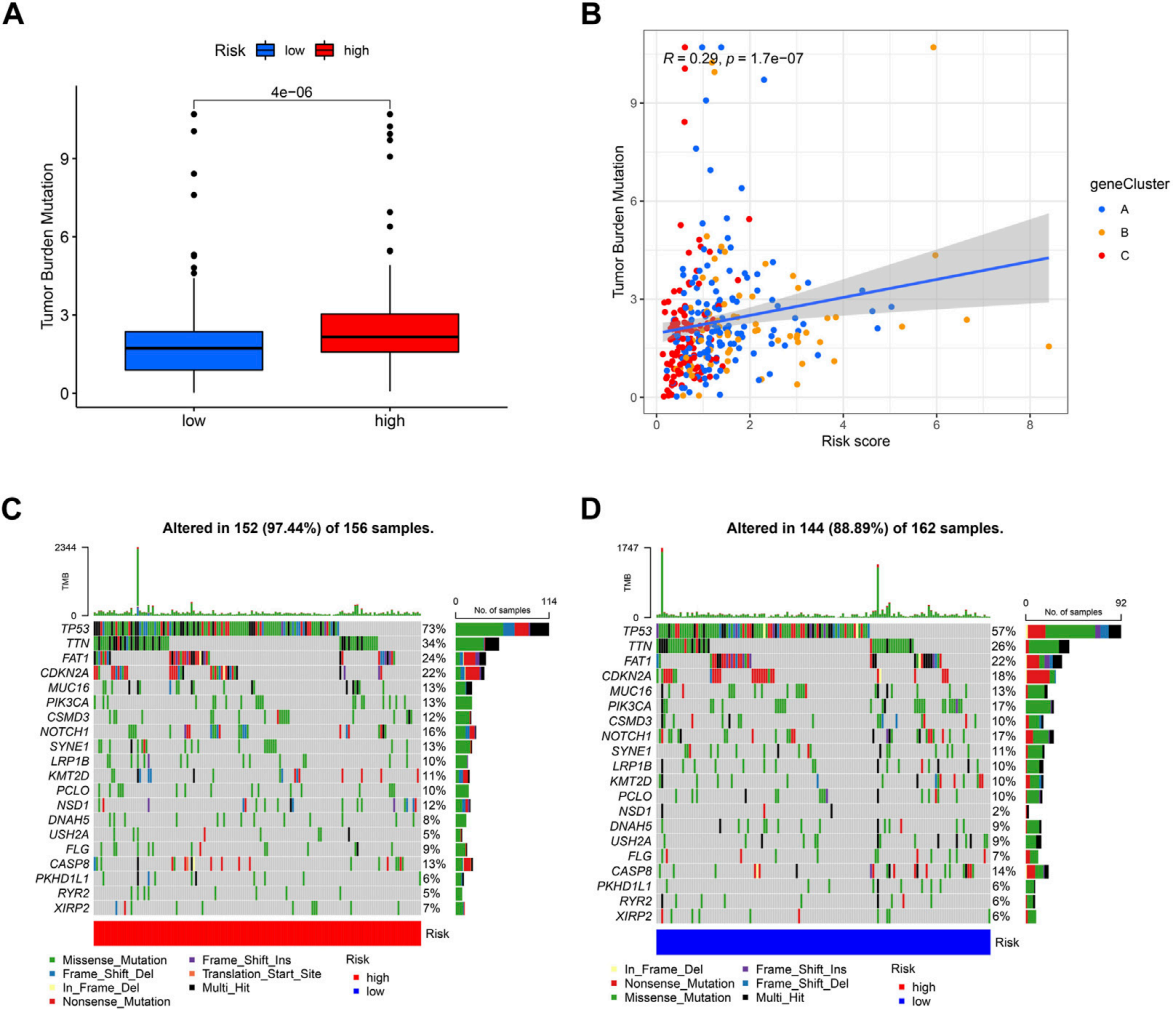
Exploration of risk scores and tumor mutation burden. **(A)** High-risk score tumors were markedly correlated with a higher TMB (p = 4e−06, Student’s *t*-test). **(B)** There was a positive correlation between risk scores and TMB (*p* = 1.7e−07). Tumor mutation landscape in high-risk **(C)** and low-risk **(D)** score groups of OSCC patients were presented in the waterfall plots.

Different risk scores are related to the TMB (tumor mutation burden) of OSCC patients. The higher the risk score of patients, the higher their TMB is (Figure 10A). Additionally, A, B, and C groups are shown to be correlated with risk scores and TMB; group C has the lowest risk score, and its TMB is lower than that of groups A and B (Figure 10B). Then, we analyzed the mutation load of different risk groups. Most of the mutations are concentrated in *TP53*, *TTN*, and *FAT1*; overall, the high-risk group had a greater mutation rate (97.44%) than the low-risk group (88.89%).

### 3.10 The IC50 of the chemotherapy between high- and low-risk groups

**FIGURE 11:**
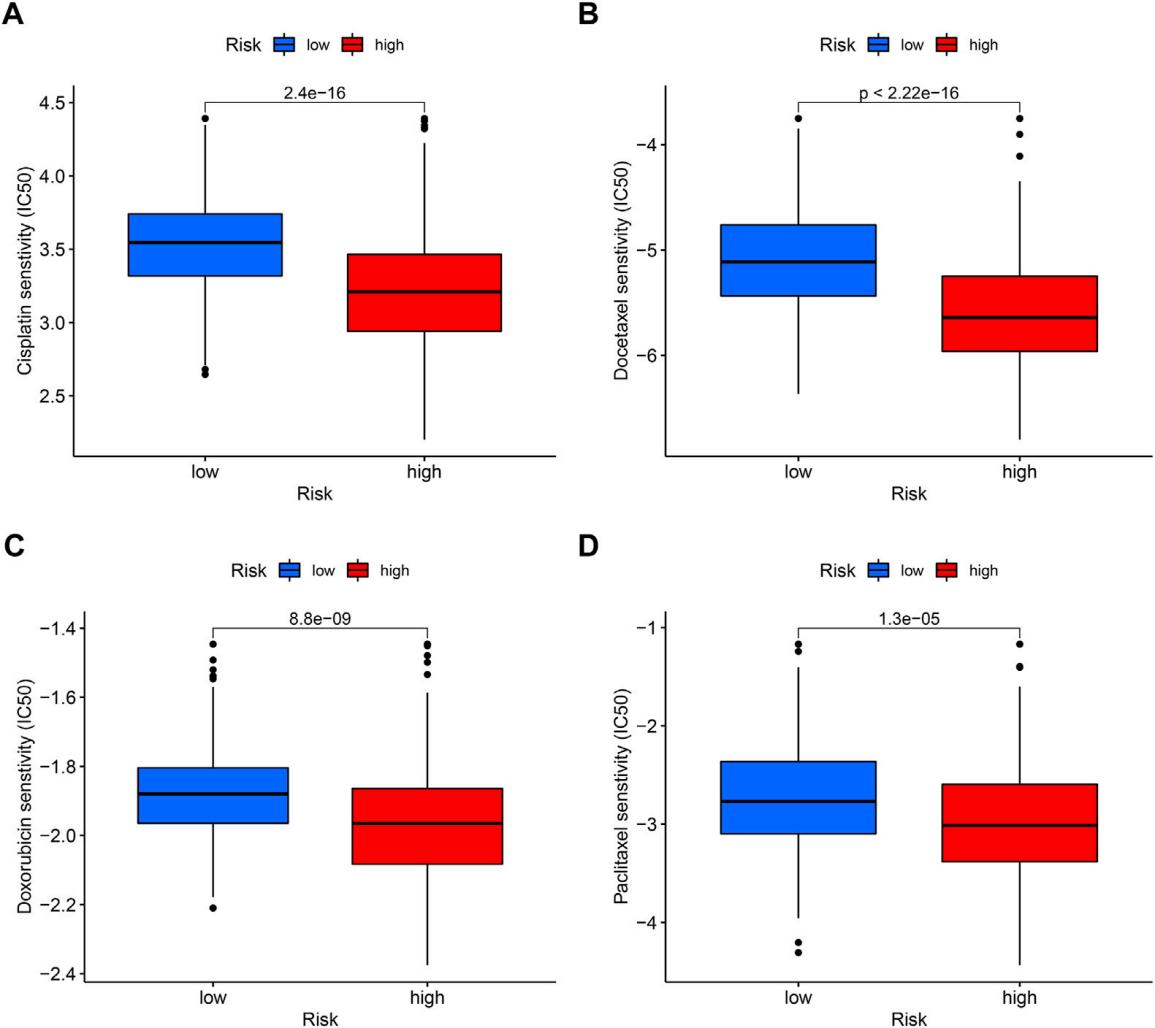
Sensitivity of low- and high-risk patients to four common chemotherapy agents. The *y*-axis represents 50% inhibitory concentration (IC50). **(A)** Cisplatin, **(B)** docetaxel, **(C)** doxorubicin, and **(D)** paclitaxel.

In tumor treatment, chemotherapy still dominates in therapeutic drugs. Depending on the risk score, it might provide various chemotherapeutic effects; a cluster with a high risk score was more likely to respond to chemotherapy. By comparing drug sensitivity, we discovered four chemotherapeutic medications, such as cisplatin, docetaxel, doxorubicin, and pacitaxel, which showed better efficacy in high-risk groups (Figures 11A–D).

### 3.11 PARDEG expression levels and its correlation between immune cell infiltration and chemotherapy

**FIGURE 12:**
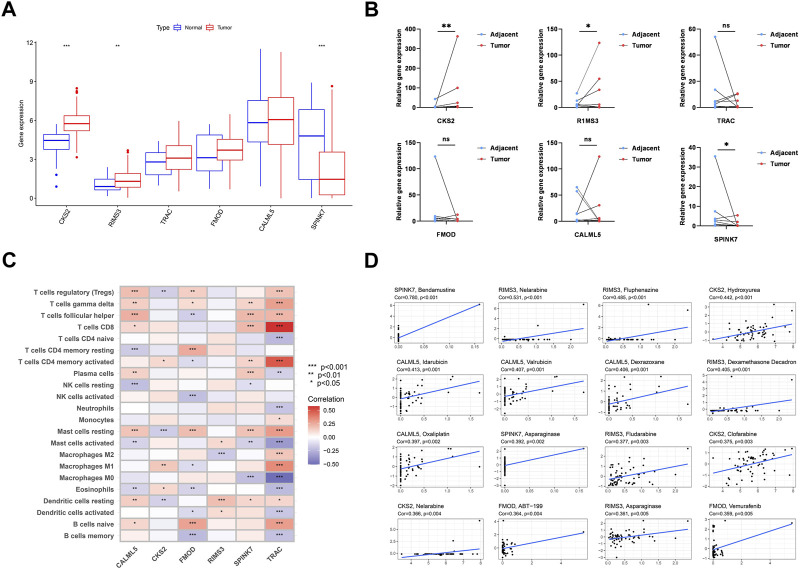
Six genes’ expression and its correlation with immune cells and chemotherapy. **(A)** Expression of six genes constructed the model. **(B)** RNA expression level of six genes in 12 pairs of tumor and normal adjacent tissues from OSCC patients. **(C)** Correlation between each TME infiltration cell 22 type and each regulator constructing the model using Spearman’s analysis. **(D)** Top 16 chemotherapy correlated to the six genes.

We examined the expression levels of PARDEG expression in cancer and normal tissues. Three genes were differently expressed between normal and tumor tissue samples in boxplots (*p* < 0.05) (Figure 12A). Two of these, *CKS2* and *RIMS3*, were shown to be substantially expressed in tumor tissues. Also, *SPINK7* is highly expressed in normal tissues. When compared to paired adjacent normal tissues in clinically collected samples, q-PCR results showed that *CKS2* was statistically substantially more expressed in the OSCC tissues (*p* < 0.001) (Figure 12B).

The heatmap shows the interaction of six genes and immune cell infiltration in OSCC. *TRAC* is significantly correlated with CD8^+^, CD4 memory-activated B cells, and macrophages (Figure 12C). Here, the association between PARDEGs and different chemotherapeutic drugs is carried out for six genes (Figure 12D), and the 16 drugs in different genes with the highest correlation are listed as follows: *SPINK7*-bendamustine, *RIMS3*-nelarabine, *CKS2*-hydroxyurea, *CALML5*-idarubicin, and FMOD-ABT-199.

## 4 Discussion

The role of the polyamine metabolism in tumors has been gradually reported, and some reviews have emerged and stated the current status of the polyamine metabolism in tumors (McNamara et al., 2021; Novita Sari et al., 2021). Lewis et al. assessed the 2-difluoromethylornithine (DFMO) as a treatment for OSCC in a cat, which is a polyamine inhibitor (Lewis et al., 2013). Polyamine-related genes might provide an exploitable treatment strategy, advance individual therapy, and enhance the prognosis of patients. Data from Chen (Hsu et al., 2019) demonstrated an overabundance of polyamine and its associated metabolites in OSCC. Ornithine decarboxylase 1 (*ODC1*), the mutations of which were concentrated in our research the most (Figure 2B **)**, is now frequently increased in cancer cells (Choi et al., 2016) and necessary for cell proliferation, transforms ornithine into putrescine, and is associated with cellular mechanisms that lead to an increase in polyamine metabolites (Miller-Fleming et al., 2015). A promising approach for overcoming the limitations of systemic therapies and advancing the science of immunotherapy is the targeting of polyamines. Therefore, it is important to properly understand and evaluate the links and processes among polyamines, immunity, and OSCC.

Amine oxidase copper containing 1 (*AOC1*) catalyzes the breakdown of related molecules and oxidatively deaminates putrescine and histamine. According to recent research studies (Ding et al., 2022), in order to stop the growth of cancer cells, the activity of *AOC1* on spermidine produces reactive oxygen species and results in ferroptosis. Proline and polyamides are produced by urea cycle enzyme arginase-1 (*ARG1*), which is essential for tumor cell growth. It is an important regulator of both innate and adaptive immune responses in the arginine metabolism. When the cell dies, it is released from the phagolysosome and depletes the microenvironment of arginine, which inhibits the proliferation of T cells, natural killer cells, and the release of cytokines (Munder et al., 2006). Consistent with this, the upregulation of *AOC1* and *ARG1* genes in OSCC was observed in our independent dataset from TCGA.

Then, to evaluate the pattern of polyamine alteration in OSCC patients, the risk score method was created by six genes as follows: “*CKS2*” (Gao et al., 2021), a subunit of the cyclin dependent kinases and is essential for their biological function. “*RIMS3*” (Deng et al., 2022) is essential for transmembrane transporter binding activity. “TRAC” (Long et al., 2021) is a protein coding gene related to immunodeficiency. “*FMOD*” (Silva et al., 2022) can affect the rate of fibrils formation as a biomarker of prostate cancer. “*CALML5*” (Kitazawa et al., 2021), which can bind calcium, may be involved in terminal differentiation of keratinocytes. The expression changes of “*SPINK7*” (Pennacchiotti et al., 2021) can be relevant in predicting OSCC at a molecular level. Patients with high-risk scores have the worse OS related to poor survival prognosis and high TMB than those with low risk scores and inhibitory TME status. These findings suggested that the risk score can offer an innovative approach to assessing the TME status and prognosis of oral squamous cell carcinoma.

The adaptive immune system’s lymphogenesis and T-cell and B-cell activation are both influenced by polyamines. One of the many ways that a B-cell activity can stop the formation of cancers is through stimulating CD4+ T cells and CD8+ T cells, as well as by producing antibodies that are reactive to tumors. Previous research has demonstrated (Zeng et al., 2021) that many subtypes have varying immunological and immune cell infiltration, which affect their prognoses and responsiveness to immunotherapy. Therefore, based on the expression of genes associated to polyamine clusters, we classified patients into three clusters. The expression of polyamine synthesis enzymes is higher in malignant tumors than in normal tissues, and this is associated with an immunosuppressive phenotype. The exchange of materials and energy between tumor cells occurs in the TME, which is crucial to the biology of tumors (Yoshihara et al., 2013). Immunosuppressive environments enable the use of polyamine-reduction methods to boost the antitumor immune responses.

In addition (Cerqueira et al., 2020), tumors evade the immune system by lowering the activity of CD8^+^ T cells while boosting the activity of CD4^+^ T cells. Our ssGSEA (Figure 5D) revealed that the expression level of CD8^+^ T cells was substantially lower in cluster A, which has a high CD4/CD8 ratio. Also, immunosuppressive myeloid-derived suppressor cells (MDSCs) are often found in abundance in the immunosuppressive microenvironment of tumors (Latour et al., 2020). When the findings of our investigation were merged with the findings of the OS analysis, they agreed with previous conclusions. As a result, we expected that polyamine-related modification patterns would alter the TME of OSCC, hence influencing survival rates. In the experimental autoimmune mouse model, it showed that the coordinated blocking of polyamine uptake and synthesis prevented T-cell-mediated inflammation (Wu et al., 2020; Chia et al., 2022). Figure 12C shows that in the foundation of the foregoing prognostic model, the *TRAC* gene mostly promotes tumor growth in OSCC patients by regulating T cells in several directions (upregulating CD8, driving naive CD4 T cells to convert to activated CD4 T cells, and inducing macrophages M0 to convert to macrophages M1 and M2).

Thus, T-cell-targeting immunotherapies and other immunotherapeutic medications can be used to treat patients. By releasing PRF1, GNLY, or GZM and rupturing previous immunological tolerance, CD8 + T cells can destroy cancer cells and improve immunotherapy by activating the PD-1/PD-L1 immune inhibitory axis. This will advance not only immunotherapy but also the study of cancer.

In addition, patients with OSCC in the high-risk group, which mainly come from cluster A (with higher polyamine-related genes expression), were more sensitive to chemotherapy drugs (Figure 11). Patients with high polyamine-related genes expressed in liquid biopsies could be distinguished by imaging mass cytometry or other experiments, which can guide through the application of chemotherapy medications. Patients with OSCC currently undergo cisplatin, paclitaxel, and docetaxel as their first-line treatments (Meng et al., 2021). Doxorubicin was usually used in combination with radiotherapy as 2B chemotherapy, to treat head and neck adenomas and metastatic tumors, according to the NCCN guidelines.

Although we had used various techniques to strengthen our model, there were still some flaws and weaknesses. Because it was a retrospective study, it was subjected to the biases that were built by this research paradigm. It was challenging to conduct external validation for prediction though we had completed internal validation in the model. The immune cell infiltration and the fitting of chemotherapy methods displayed the results from several platforms, and we also used collected tissues to validate the RNA expression level, which could be viewed as external validation in a certain sense. We intend to gather more clinical datasets to reaffirm the importance of these polyamine-related genes.

## 5 Conclusion

In this study, two distinct polyamine modification patterns were discovered, and the immune cell infiltration was different in these clusters, indicating that the modification played a vital role in shaping individual tumor microenvironment characterizations. We built a six PARDEG model and attempted to identify the prognosis and immune infiltration of OSCCs. Moreover, the model could assess individual tumor polyamine alteration patterns and predict the clinical response of patients to chemical therapy. These findings are conducive to understanding the mechanism of polyamine in OSCC and provide novel targets for treating OSCC patients.

## Data Availability

Publicly available datasets were analyzed in this study. These data can be found online in the following: the datasets involved during the current study are available in TCGA (https://portal.gdc.cancer.gov/) and GEO (https://www.ncbi.nlm.nih.gov/gds/), and the protein–protein interaction (PPI) database was from STRING (https://string-db.org/).
