# Supplementary material for: Identification of a polyamine-related signature and six novel prognostic biomarkers in oral squamous cell carcinoma
Source: Front Mol Biosci. 2023 Jan 17;10:1073770. doi: 10.3389/fmolb.2023.1073770 (PMC9887031; doi:10.3389/fmolb.2023.1073770)
Supplement: Supplementary file 1 [file DataSheet1.docx]

Supplementary Material

# Supplementary Figures


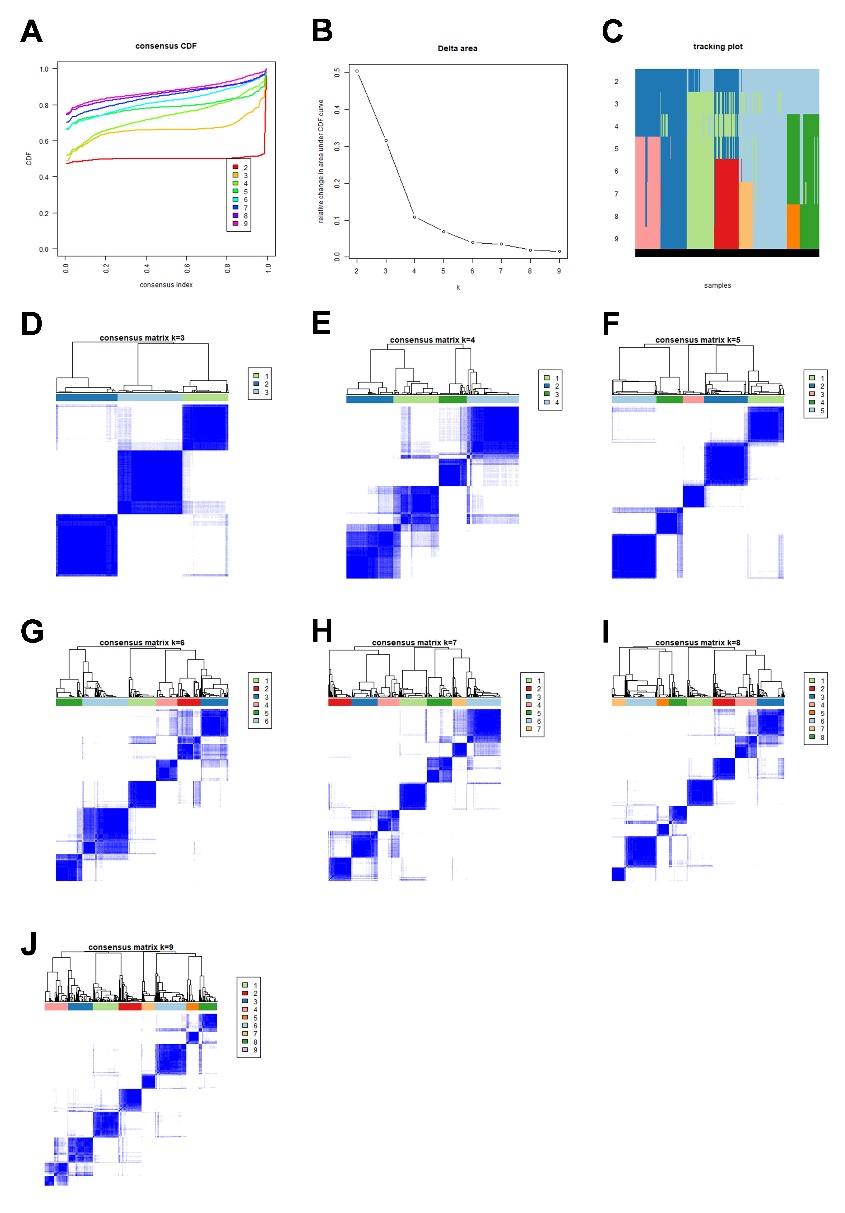


**Figure S1**

(A) Consensus clustering distribution function (CDF) for k = 2-9 (B) Area under CDF curve increment for k = 2-9. (C) Tracking plot for k = 2-9.

(D-J) Consensus clustering matrix for 17 polyamine related genes in OSCC samples for k = 3-9.


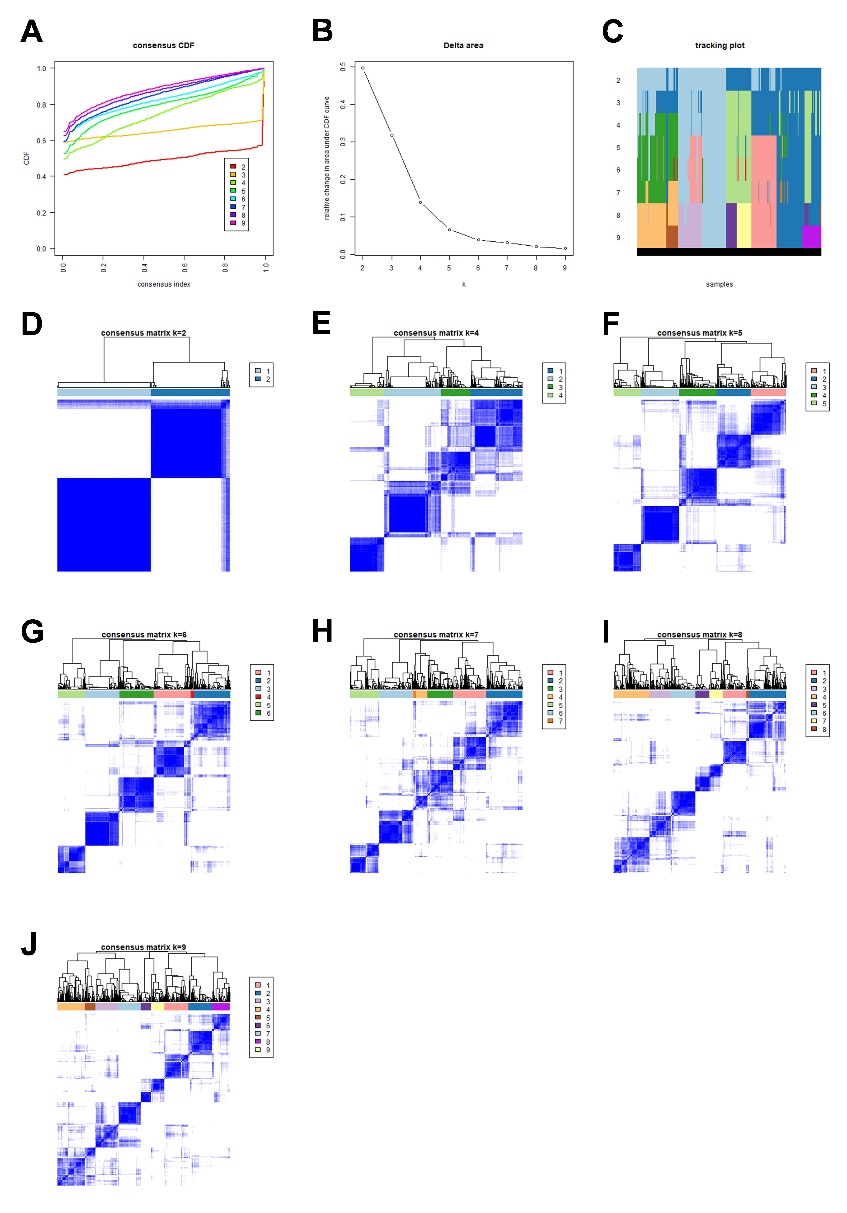


**Figure S2**

(A) Consensus clustering distribution function (CDF) for k = 2-9. (B) Area under CDF curve increment for k = 2-9. (C) Tracking plot for k = 2-9.

(D-J) Identification of consensus clusters by DEGs related to PRGs clusters. Consensus clustering matrix for k = 2, k = 4-9.

# Supplementary Tables

**Table S1**. Differentially expressed genes (DEGs) between the two PARG clusters

| Genes | logFC | AveExpr | t | P.Value | adj.P.Val | B |
| --- | --- | --- | --- | --- | --- | --- |
| AOC1 | 1.837833 | 2.436389 | 28.71973 | 4.36E-101 | 7.69E-97 | 213.4446 |
| CD1A | 1.083348 | 2.913672 | 8.582479 | 1.83E-16 | 1.07E-12 | 26.70689 |
| CCL22 | 0.877134 | 3.675897 | 8.536605 | 2.56E-16 | 1.09E-12 | 26.38017 |
| FCER1A | 1.040939 | 2.920772 | 8.309825 | 1.34E-15 | 2.96E-12 | 24.78311 |
| CD1E | 0.701979 | 1.81844 | 8.073839 | 7.28E-15 | 1.07E-11 | 23.15377 |
| S100B | 0.800738 | 3.62032 | 7.81093 | 4.60E-14 | 3.86E-11 | 21.37879 |
| NUF2 | -0.6589 | 4.51301 | -7.79543 | 5.12E-14 | 4.06E-11 | 21.2755 |
| CKS2 | -0.63393 | 8.018018 | -7.59322 | 2.05E-13 | 1.39E-10 | 19.94199 |
| DGAT2 | 0.820601 | 3.241823 | 7.555664 | 2.64E-13 | 1.72E-10 | 19.69718 |
| HLA-DQB2 | 1.146201 | 4.864631 | 7.476579 | 4.50E-13 | 2.41E-10 | 19.18473 |
| AQP3 | 1.309365 | 9.22008 | 7.475789 | 4.52E-13 | 2.41E-10 | 19.17963 |
| BLNK | 0.79408 | 4.48049 | 7.40631 | 7.19E-13 | 3.46E-10 | 18.73284 |
| KAT2B | 0.596983 | 4.199045 | 7.404769 | 7.27E-13 | 3.46E-10 | 18.72297 |
| UBE2T | -0.6331 | 5.790271 | -7.37227 | 9.02E-13 | 4.07E-10 | 18.5151 |
| CD1C | 0.659708 | 2.290901 | 7.293157 | 1.52E-12 | 6.24E-10 | 18.01204 |
| MND1 | -0.58557 | 3.474944 | -7.26451 | 1.84E-12 | 7.36E-10 | 17.83088 |
| CSF2RB | 0.795089 | 4.284369 | 7.253357 | 1.98E-12 | 7.74E-10 | 17.76053 |
| KRT1 | 2.465538 | 7.489776 | 7.238905 | 2.17E-12 | 8.33E-10 | 17.66947 |
| PCDH1 | 0.606313 | 4.360035 | 7.199759 | 2.81E-12 | 1.05E-09 | 17.42352 |
| KCND3 | 0.608932 | 2.368952 | 7.171754 | 3.37E-12 | 1.21E-09 | 17.24819 |
| PLA2G2F | 0.766096 | 1.74867 | 7.155394 | 3.75E-12 | 1.29E-09 | 17.14602 |
| SDR9C7 | 1.337489 | 4.452971 | 7.1531 | 3.81E-12 | 1.29E-09 | 17.13171 |
| CYP7B1 | 0.71831 | 2.677999 | 7.097075 | 5.47E-12 | 1.66E-09 | 16.78327 |
| AURKB | -0.60772 | 5.30134 | -7.07484 | 6.32E-12 | 1.80E-09 | 16.6456 |
| PBK | -0.64589 | 4.954109 | -7.03694 | 8.06E-12 | 2.22E-09 | 16.41165 |
| CCR7 | 0.843881 | 3.180603 | 7.034311 | 8.19E-12 | 2.22E-09 | 16.39547 |
| CD207 | 1.06662 | 3.173546 | 6.995056 | 1.05E-11 | 2.69E-09 | 16.1543 |
| RFC4 | -0.58784 | 5.618272 | -6.98226 | 1.14E-11 | 2.85E-09 | 16.07594 |
| KRT10 | 1.445315 | 8.895854 | 6.981315 | 1.15E-11 | 2.85E-09 | 16.07013 |
| CST6 | 1.182719 | 5.768346 | 6.930555 | 1.59E-11 | 3.46E-09 | 15.76033 |
| GAS7 | 0.657843 | 3.307345 | 6.904204 | 1.88E-11 | 3.84E-09 | 15.6002 |
| FCGBP | 1.027852 | 3.439489 | 6.885338 | 2.11E-11 | 4.28E-09 | 15.48586 |
| ALDH3B2 | 1.061729 | 6.34968 | 6.875596 | 2.25E-11 | 4.46E-09 | 15.42692 |
| SLC24A3 | 0.81556 | 4.406026 | 6.853986 | 2.58E-11 | 4.99E-09 | 15.29639 |
| BPIFC | 1.018858 | 2.329563 | 6.852172 | 2.61E-11 | 4.99E-09 | 15.28545 |
| RDH16 | 0.68224 | 3.095678 | 6.821135 | 3.17E-11 | 5.81E-09 | 15.0986 |
| LYNX1 | 1.056822 | 4.994743 | 6.776079 | 4.20E-11 | 7.04E-09 | 14.82855 |
| CCDC69 | 0.591576 | 4.26927 | 6.745034 | 5.09E-11 | 7.86E-09 | 14.64332 |
| CLEC10A | 0.7072 | 2.798227 | 6.66232 | 8.49E-11 | 1.13E-08 | 14.1531 |
| CD5 | 0.622787 | 2.700815 | 6.558895 | 1.60E-10 | 1.93E-08 | 13.547 |
| TMEM86A | 0.590601 | 3.067125 | 6.541316 | 1.78E-10 | 2.10E-08 | 13.44475 |
| MYO1G | 0.588728 | 3.332693 | 6.425018 | 3.58E-10 | 3.63E-08 | 12.77388 |
| NAPSB | 0.711896 | 3.271847 | 6.379887 | 4.69E-10 | 4.52E-08 | 12.51618 |
| STX11 | 0.621186 | 3.813706 | 6.338175 | 6.01E-10 | 5.49E-08 | 12.27933 |
| P2RY13 | 0.606719 | 2.164269 | 6.326754 | 6.43E-10 | 5.72E-08 | 12.2147 |
| TMPRSS13 | 0.67284 | 4.133727 | 6.312061 | 7.01E-10 | 6.12E-08 | 12.1317 |
| KRT16 | 1.192196 | 12.63171 | 6.305299 | 7.30E-10 | 6.27E-08 | 12.09355 |
| EPCAM | -1.0171 | 5.924964 | -6.27861 | 8.54E-10 | 7.10E-08 | 11.94334 |
| DSG1 | 1.698795 | 6.695829 | 6.264596 | 9.27E-10 | 7.56E-08 | 11.86465 |
| S100A7A | 1.461575 | 4.86408 | 6.248933 | 1.02E-09 | 8.14E-08 | 11.77688 |
| RIMS3 | 0.598334 | 2.847954 | 6.244495 | 1.04E-09 | 8.28E-08 | 11.75205 |
| DSC2 | 0.8393 | 7.677147 | 6.241206 | 1.06E-09 | 8.40E-08 | 11.73366 |
| IL2RB | 0.727514 | 4.133094 | 6.237264 | 1.09E-09 | 8.52E-08 | 11.71162 |
| SLC39A2 | 0.883467 | 3.877601 | 6.229092 | 1.14E-09 | 8.78E-08 | 11.66598 |
| CWH43 | 1.201245 | 3.446072 | 6.196274 | 1.38E-09 | 1.02E-07 | 11.48317 |
| C10orf99 | 1.373415 | 7.978739 | 6.184383 | 1.48E-09 | 1.07E-07 | 11.41713 |
| LTB | 0.623876 | 3.829698 | 6.163731 | 1.67E-09 | 1.19E-07 | 11.30268 |
| TGM5 | 0.769713 | 3.019735 | 6.084623 | 2.63E-09 | 1.68E-07 | 10.86724 |
| RDH12 | 1.147363 | 3.491603 | 6.076987 | 2.75E-09 | 1.75E-07 | 10.82546 |
| TMEM79 | 0.66333 | 6.873725 | 6.033671 | 3.52E-09 | 2.17E-07 | 10.58927 |
| ARL14 | 0.856103 | 2.947016 | 5.998701 | 4.30E-09 | 2.55E-07 | 10.39961 |
| LYPD5 | 0.832031 | 4.48023 | 5.984244 | 4.67E-09 | 2.72E-07 | 10.32148 |
| SPINK5 | 1.262345 | 6.533316 | 5.977711 | 4.84E-09 | 2.81E-07 | 10.28622 |
| RASSF2 | 0.596866 | 3.804345 | 5.974128 | 4.94E-09 | 2.84E-07 | 10.2669 |
| RASGRP1 | 0.669864 | 3.514294 | 5.955314 | 5.50E-09 | 3.09E-07 | 10.1656 |
| SLURP1 | 1.635171 | 6.029081 | 5.952244 | 5.59E-09 | 3.12E-07 | 10.14909 |
| SLC28A3 | 0.704692 | 4.038764 | 5.944627 | 5.84E-09 | 3.22E-07 | 10.10818 |
| TMEM45A | 0.798541 | 6.595787 | 5.931013 | 6.30E-09 | 3.46E-07 | 10.03515 |
| USP2 | 0.600656 | 2.921918 | 5.877665 | 8.49E-09 | 4.40E-07 | 9.750358 |
| BBOX1 | 0.837496 | 3.55697 | 5.855348 | 9.62E-09 | 4.79E-07 | 9.631864 |
| CST7 | 0.659274 | 4.623594 | 5.824699 | 1.14E-08 | 5.47E-07 | 9.469754 |
| ANKRD22 | 0.679739 | 5.60634 | 5.815202 | 1.20E-08 | 5.64E-07 | 9.419666 |
| TRAC | 0.742237 | 5.272751 | 5.802863 | 1.29E-08 | 5.98E-07 | 9.354695 |
| CDSN | 1.09003 | 2.996268 | 5.799115 | 1.31E-08 | 6.08E-07 | 9.334984 |
| HES2 | 0.605962 | 5.164141 | 5.788158 | 1.40E-08 | 6.35E-07 | 9.277419 |
| CXCR3 | 0.618882 | 2.444315 | 5.750558 | 1.72E-08 | 7.63E-07 | 9.080587 |
| PDZK1IP1 | 0.949712 | 7.808643 | 5.708389 | 2.16E-08 | 9.10E-07 | 8.861132 |
| SLCO2A1 | 0.676114 | 4.789044 | 5.699934 | 2.26E-08 | 9.44E-07 | 8.817294 |
| CD36 | 0.657482 | 2.829953 | 5.698349 | 2.28E-08 | 9.50E-07 | 8.809083 |
| LCE3D | 1.551808 | 7.519355 | 5.672703 | 2.62E-08 | 1.03E-06 | 8.676493 |
| DIO2 | 0.77199 | 4.596103 | 5.633112 | 3.25E-08 | 1.23E-06 | 8.472808 |
| WNT4 | 0.648569 | 4.036364 | 5.627641 | 3.35E-08 | 1.26E-06 | 8.444755 |
| SELL | 0.732998 | 3.720461 | 5.616063 | 3.56E-08 | 1.32E-06 | 8.385467 |
| CD2 | 0.787844 | 4.817886 | 5.594684 | 3.99E-08 | 1.46E-06 | 8.276271 |
| PLA2G4F | 0.663714 | 3.059982 | 5.579685 | 4.33E-08 | 1.56E-06 | 8.199875 |
| SLAMF7 | 0.651923 | 4.152033 | 5.552634 | 5.00E-08 | 1.74E-06 | 8.062537 |
| KRT2 | 1.116364 | 1.985401 | 5.551818 | 5.02E-08 | 1.75E-06 | 8.058404 |
| BNIPL | 0.760587 | 4.207232 | 5.548422 | 5.12E-08 | 1.77E-06 | 8.041203 |
| CHI3L2 | 0.795166 | 3.078765 | 5.544855 | 5.21E-08 | 1.79E-06 | 8.023147 |
| SAMD9 | 0.684711 | 6.97654 | 5.528039 | 5.70E-08 | 1.92E-06 | 7.938166 |
| KRT80 | 0.851315 | 6.092837 | 5.510559 | 6.26E-08 | 2.07E-06 | 7.850061 |
| STEAP4 | 0.829615 | 3.743318 | 5.492005 | 6.90E-08 | 2.25E-06 | 7.756812 |
| WFDC12 | 1.386899 | 4.564762 | 5.469671 | 7.76E-08 | 2.46E-06 | 7.64492 |
| IVL | 1.096395 | 8.467754 | 5.417699 | 1.02E-07 | 3.07E-06 | 7.386069 |
| APCDD1 | 0.588754 | 5.99951 | 5.416839 | 1.02E-07 | 3.08E-06 | 7.381804 |
| IL2RG | 0.660788 | 5.134153 | 5.412077 | 1.05E-07 | 3.13E-06 | 7.358196 |
| SPNS2 | 0.643625 | 4.076391 | 5.406329 | 1.08E-07 | 3.20E-06 | 7.329725 |
| CD3E | 0.651802 | 3.64539 | 5.372695 | 1.29E-07 | 3.70E-06 | 7.163658 |
| IRF8 | 0.589211 | 3.736572 | 5.369218 | 1.31E-07 | 3.75E-06 | 7.146539 |
| CD27 | 0.689355 | 3.799988 | 5.355301 | 1.41E-07 | 3.98E-06 | 7.078124 |
| PTPRC | 0.661796 | 4.100672 | 5.354821 | 1.41E-07 | 3.98E-06 | 7.075769 |
| DMKN | 0.89853 | 7.104849 | 5.354581 | 1.42E-07 | 3.98E-06 | 7.07459 |
| PGLYRP4 | 0.820973 | 4.056572 | 5.352259 | 1.43E-07 | 4.02E-06 | 7.063192 |
| WFDC5 | 0.906943 | 5.409691 | 5.337786 | 1.54E-07 | 4.24E-06 | 6.992241 |
| VSIG10L | 0.884729 | 4.673875 | 5.304834 | 1.83E-07 | 4.82E-06 | 6.83133 |
| MUC15 | 0.846094 | 3.234265 | 5.286467 | 2.01E-07 | 5.21E-06 | 6.742017 |
| KRT6B | 0.956206 | 12.10831 | 5.285826 | 2.02E-07 | 5.22E-06 | 6.738908 |
| SERPINB7 | 0.917553 | 4.433976 | 5.271967 | 2.17E-07 | 5.50E-06 | 6.671699 |
| CCR5 | 0.626007 | 3.336876 | 5.25522 | 2.36E-07 | 5.88E-06 | 6.590694 |
| RAET1E | 0.706082 | 3.496867 | 5.252918 | 2.39E-07 | 5.93E-06 | 6.579578 |
| FMOD | 0.619337 | 6.037166 | 5.251058 | 2.41E-07 | 5.98E-06 | 6.570598 |
| GJB2 | 0.831129 | 10.66879 | 5.242301 | 2.52E-07 | 6.21E-06 | 6.528359 |
| ACER1 | 0.613216 | 1.677057 | 5.237416 | 2.58E-07 | 6.34E-06 | 6.504823 |
| S100A7 | 1.355546 | 11.28115 | 5.227088 | 2.72E-07 | 6.62E-06 | 6.455127 |
| PNLIPRP3 | 1.052001 | 3.7125 | 5.20402 | 3.06E-07 | 7.25E-06 | 6.344437 |
| HLA-DRB6 | 0.731837 | 4.974349 | 5.193764 | 3.22E-07 | 7.54E-06 | 6.29536 |
| NSG1 | 0.693237 | 4.600986 | 5.19297 | 3.23E-07 | 7.57E-06 | 6.291564 |
| DSC1 | 1.204959 | 3.804077 | 5.187246 | 3.33E-07 | 7.73E-06 | 6.264218 |
| SLC6A14 | 0.973454 | 4.162948 | 5.178985 | 3.47E-07 | 7.97E-06 | 6.224792 |
| PPP2R2C | 0.665855 | 4.782522 | 5.174998 | 3.54E-07 | 8.10E-06 | 6.205785 |
| CNFN | 1.194684 | 9.452949 | 5.164324 | 3.74E-07 | 8.42E-06 | 6.154962 |
| SORCS2 | 0.620669 | 3.728173 | 5.152276 | 3.97E-07 | 8.82E-06 | 6.097709 |
| SERPINB12 | 0.69069 | 1.633339 | 5.136464 | 4.30E-07 | 9.36E-06 | 6.022746 |
| KRT8 | -1.08209 | 6.420756 | -5.11828 | 4.70E-07 | 1.01E-05 | 5.936806 |
| SERPINB4 | 1.148291 | 6.048597 | 5.113005 | 4.83E-07 | 1.03E-05 | 5.911904 |
| ZNF185 | 0.600894 | 7.381132 | 5.105452 | 5.02E-07 | 1.05E-05 | 5.876312 |
| ABCA12 | 0.764207 | 4.306235 | 5.075679 | 5.81E-07 | 1.19E-05 | 5.736458 |
| LYPD3 | 0.647799 | 9.409454 | 5.065997 | 6.10E-07 | 1.24E-05 | 5.691136 |
| KLK7 | 1.139633 | 6.786668 | 5.027402 | 7.38E-07 | 1.46E-05 | 5.511224 |
| KRT18 | -0.63551 | 7.532911 | -5.01659 | 7.78E-07 | 1.52E-05 | 5.461051 |
| SLC22A3 | 0.679733 | 3.937381 | 5.015508 | 7.82E-07 | 1.53E-05 | 5.456022 |
| FOXN1 | 0.680337 | 4.091382 | 5.002089 | 8.36E-07 | 1.61E-05 | 5.393885 |
| SCNN1B | 0.739659 | 2.890827 | 5.000983 | 8.40E-07 | 1.62E-05 | 5.388769 |
| HLA-DPB1 | 0.633938 | 7.060687 | 4.96358 | 1.01E-06 | 1.89E-05 | 5.216384 |
| ENDOU | 0.857113 | 2.862739 | 4.956229 | 1.04E-06 | 1.94E-05 | 5.18264 |
| LIMCH1 | 0.596732 | 3.373183 | 4.95313 | 1.06E-06 | 1.97E-05 | 5.168429 |
| TPSAB1 | 0.627818 | 4.264018 | 4.940973 | 1.13E-06 | 2.06E-05 | 5.112747 |
| POU3F1 | 0.639606 | 3.497387 | 4.93959 | 1.13E-06 | 2.06E-05 | 5.106421 |
| MFAP4 | 0.822455 | 4.411949 | 4.937272 | 1.15E-06 | 2.08E-05 | 5.095821 |
| CALML5 | 1.216948 | 7.423973 | 4.891376 | 1.43E-06 | 2.49E-05 | 4.886856 |
| RAC3 | -0.59183 | 3.961706 | -4.8738 | 1.56E-06 | 2.65E-05 | 4.807279 |
| CLIC3 | 0.757195 | 6.568927 | 4.866338 | 1.61E-06 | 2.74E-05 | 4.77359 |
| ELOVL4 | 0.69892 | 3.875036 | 4.865111 | 1.62E-06 | 2.75E-05 | 4.768051 |
| SBSN | 1.183591 | 9.317803 | 4.859427 | 1.67E-06 | 2.82E-05 | 4.742414 |
| HLA-DRA | 0.665698 | 10.4754 | 4.859058 | 1.67E-06 | 2.82E-05 | 4.740753 |
| S100A9 | 0.799233 | 13.81949 | 4.848128 | 1.76E-06 | 2.95E-05 | 4.691535 |
| LCE2B | 0.893346 | 2.022568 | 4.834483 | 1.88E-06 | 3.10E-05 | 4.630231 |
| LYZ | 0.864275 | 7.770383 | 4.815961 | 2.05E-06 | 3.32E-05 | 4.547259 |
| CD74 | 0.650058 | 9.39639 | 4.777951 | 2.45E-06 | 3.84E-05 | 4.377888 |
| FGL2 | 0.613535 | 4.69735 | 4.754776 | 2.74E-06 | 4.22E-05 | 4.27521 |
| SPRR2G | 1.433619 | 8.085548 | 4.745683 | 2.85E-06 | 4.36E-05 | 4.235042 |
| KLK6 | 1.120318 | 6.236313 | 4.726067 | 3.13E-06 | 4.72E-05 | 4.148631 |
| KRT6A | 0.644855 | 13.62427 | 4.725975 | 3.13E-06 | 4.72E-05 | 4.148225 |
| CCL24 | 0.599719 | 2.756703 | 4.721234 | 3.20E-06 | 4.81E-05 | 4.127388 |
| ANKRD35 | 0.62129 | 3.630773 | 4.715233 | 3.29E-06 | 4.92E-05 | 4.101044 |
| FPR3 | 0.593788 | 4.556279 | 4.712585 | 3.33E-06 | 4.96E-05 | 4.089428 |
| WNT10A | 0.603098 | 3.784214 | 4.708828 | 3.39E-06 | 5.04E-05 | 4.072953 |
| ZNF750 | 0.674928 | 6.612439 | 4.698771 | 3.55E-06 | 5.23E-05 | 4.028922 |
| PGLYRP3 | 0.668705 | 4.430495 | 4.695489 | 3.61E-06 | 5.28E-05 | 4.014569 |
| CRABP2 | 0.646655 | 8.806069 | 4.678198 | 3.91E-06 | 5.65E-05 | 3.939107 |
| PLEK | 0.602033 | 4.496063 | 4.670685 | 4.05E-06 | 5.82E-05 | 3.906393 |
| SLC5A1 | 0.684552 | 2.706023 | 4.653778 | 4.38E-06 | 6.17E-05 | 3.832955 |
| PRELP | 0.654568 | 2.747031 | 4.648852 | 4.48E-06 | 6.27E-05 | 3.811603 |
| HLA-DPA1 | 0.677095 | 7.159646 | 4.632666 | 4.82E-06 | 6.68E-05 | 3.741588 |
| SERPINB3 | 1.008692 | 7.500872 | 4.590521 | 5.85E-06 | 7.78E-05 | 3.560316 |
| SERPINB13 | 0.811285 | 5.681801 | 4.582196 | 6.08E-06 | 8.04E-05 | 3.524688 |
| LY6D | 0.964639 | 9.863269 | 4.564463 | 6.59E-06 | 8.54E-05 | 3.448986 |
| NIPAL4 | 0.683931 | 5.648539 | 4.56104 | 6.69E-06 | 8.67E-05 | 3.434403 |
| MMP28 | 0.631067 | 4.951549 | 4.532047 | 7.63E-06 | 9.68E-05 | 3.311285 |
| GZMK | 0.651708 | 2.899987 | 4.521219 | 8.01E-06 | 0.0001 | 3.265489 |
| IGHG1 | 0.944751 | 7.035748 | 4.498067 | 8.89E-06 | 0.00011 | 3.167898 |
| DPT | 0.725766 | 4.287049 | 4.497733 | 8.90E-06 | 0.00011 | 3.166497 |
| HAL | 0.590489 | 1.609184 | 4.496094 | 8.97E-06 | 0.00011 | 3.159606 |
| ALOX12B | 0.979351 | 4.181955 | 4.491347 | 9.16E-06 | 0.000112 | 3.13966 |
| SULT2B1 | 0.752921 | 6.0848 | 4.477122 | 9.76E-06 | 0.000118 | 3.080004 |
| SCEL | 0.817779 | 5.125433 | 4.464581 | 1.03E-05 | 0.000124 | 3.027552 |
| PPL | 0.639834 | 7.669901 | 4.427774 | 1.22E-05 | 0.000142 | 2.874386 |
| CYSRT1 | 0.803631 | 5.714718 | 4.42378 | 1.24E-05 | 0.000144 | 2.857835 |
| CPA3 | 0.639995 | 4.433998 | 4.414461 | 1.29E-05 | 0.00015 | 2.819268 |
| SPRR2B | 1.263743 | 7.401229 | 4.394529 | 1.41E-05 | 0.000161 | 2.737035 |
| FST | -0.64314 | 6.67986 | -4.3921 | 1.42E-05 | 0.000162 | 2.727029 |
| KRT78 | 0.939225 | 4.317424 | 4.371375 | 1.56E-05 | 0.000175 | 2.641929 |
| ASPRV1 | 1.057741 | 4.830282 | 4.369815 | 1.57E-05 | 0.000176 | 2.635535 |
| FLG2 | 0.713381 | 1.697922 | 4.356499 | 1.66E-05 | 0.000184 | 2.581067 |
| GJB6 | 0.816823 | 8.961967 | 4.35567 | 1.67E-05 | 0.000184 | 2.577678 |
| SPINK7 | 1.162854 | 4.126692 | 4.346184 | 1.74E-05 | 0.000191 | 2.538974 |
| SLPI | 0.711294 | 10.99467 | 4.335976 | 1.82E-05 | 0.000198 | 2.497411 |
| RHCG | 1.110404 | 7.326947 | 4.29038 | 2.22E-05 | 0.000233 | 2.312845 |
| CRCT1 | 1.079668 | 6.689992 | 4.283922 | 2.28E-05 | 0.000238 | 2.286847 |
| AKR1C1 | -0.84359 | 4.484525 | -4.27379 | 2.38E-05 | 0.000246 | 2.246122 |
| FABP4 | 1.160084 | 4.497635 | 4.257889 | 2.55E-05 | 0.00026 | 2.182413 |
| CD8A | 0.63625 | 3.5226 | 4.242391 | 2.72E-05 | 0.000275 | 2.12052 |
| PRSS27 | 0.716748 | 3.558126 | 4.231808 | 2.85E-05 | 0.000284 | 2.078373 |
| PI3 | 1.058722 | 11.81558 | 4.216309 | 3.04E-05 | 0.0003 | 2.016822 |
| TPSB2 | 0.612114 | 4.487706 | 4.216171 | 3.05E-05 | 0.0003 | 2.016277 |
| FABP5 | 0.635728 | 10.5431 | 4.199867 | 3.26E-05 | 0.000318 | 1.951753 |
| CYP4F22 | 0.764504 | 3.027321 | 4.161092 | 3.84E-05 | 0.000364 | 1.799228 |
| LRRC15 | 0.707926 | 4.019879 | 4.154009 | 3.96E-05 | 0.000373 | 1.771504 |
| HLA-DQA1 | 0.758063 | 5.313486 | 4.151888 | 4.00E-05 | 0.000375 | 1.763213 |
| SPRR1B | 0.921354 | 11.95003 | 4.147999 | 4.06E-05 | 0.00038 | 1.748016 |
| PLA2G3 | 0.656261 | 3.080976 | 4.1291 | 4.40E-05 | 0.000406 | 1.674359 |
| KLK11 | 0.737223 | 6.400552 | 4.087821 | 5.22E-05 | 0.000469 | 1.514556 |
| IGHV1-69 | 0.833919 | 3.127843 | 4.085039 | 5.28E-05 | 0.000473 | 1.50384 |
| IL36RN | 0.845184 | 5.924768 | 4.082357 | 5.34E-05 | 0.000476 | 1.493517 |
| CDA | 0.71899 | 6.488552 | 4.046389 | 6.19E-05 | 0.000541 | 1.355651 |
| CAPNS2 | 0.685188 | 6.223557 | 4.033872 | 6.52E-05 | 0.000565 | 1.307935 |
| CXCL13 | 0.705256 | 5.932063 | 4.029248 | 6.64E-05 | 0.000575 | 1.290344 |
| MMP3 | 0.910987 | 7.355236 | 4.01565 | 7.02E-05 | 0.000602 | 1.238719 |
| CYP4F11 | -0.73174 | 3.067718 | -4.01545 | 7.03E-05 | 0.000602 | 1.237957 |
| CCL21 | 0.803647 | 5.758817 | 4.003126 | 7.39E-05 | 0.000628 | 1.191315 |
| F13A1 | 0.648138 | 4.959368 | 3.996664 | 7.59E-05 | 0.00064 | 1.166911 |
| S100A8 | 0.686462 | 11.77056 | 3.984527 | 7.97E-05 | 0.000668 | 1.12117 |
| COL17A1 | 0.679667 | 8.95955 | 3.949606 | 9.18E-05 | 0.000748 | 0.990282 |
| CCL19 | 0.781295 | 4.541686 | 3.944963 | 9.35E-05 | 0.000758 | 0.972961 |
| KRTDAP | 1.166764 | 9.661238 | 3.943134 | 9.42E-05 | 0.000762 | 0.966143 |
| POF1B | 0.720411 | 4.180575 | 3.938546 | 9.60E-05 | 0.000774 | 0.949053 |
| FLG | 0.686729 | 2.280566 | 3.93817 | 9.61E-05 | 0.000775 | 0.947653 |
| CXCL9 | 0.899209 | 6.569534 | 3.937559 | 9.64E-05 | 0.000776 | 0.945376 |
| KLK13 | 0.91277 | 5.3199 | 3.916959 | 0.000105 | 0.000831 | 0.868886 |
| S100P | 0.799134 | 6.449811 | 3.911513 | 0.000107 | 0.000845 | 0.848725 |
| CD79A | 0.651469 | 3.396022 | 3.906305 | 0.000109 | 0.000858 | 0.829469 |
| KLK8 | 0.69645 | 6.322199 | 3.844657 | 0.000139 | 0.001048 | 0.603361 |
| IGLV1-44 | 0.950713 | 5.557471 | 3.844397 | 0.00014 | 0.001049 | 0.602414 |
| S100A12 | 0.822739 | 6.886667 | 3.843446 | 0.00014 | 0.001051 | 0.598953 |
| COMP | 0.801717 | 3.92211 | 3.837631 | 0.000143 | 0.001072 | 0.577803 |
| VCAM1 | 0.587164 | 3.965286 | 3.837583 | 0.000143 | 0.001072 | 0.57763 |
| RPL39L | -0.63426 | 4.873884 | -3.79308 | 0.000171 | 0.001238 | 0.416751 |
| SPRR1A | 0.971258 | 11.38523 | 3.789508 | 0.000173 | 0.001253 | 0.403925 |
| THBS4 | 0.899977 | 4.144197 | 3.74748 | 0.000204 | 0.001433 | 0.253744 |
| TGM1 | 0.769806 | 7.295327 | 3.716993 | 0.000229 | 0.00157 | 0.145783 |
| FLRT3 | 0.664629 | 4.641469 | 3.701918 | 0.000243 | 0.001648 | 0.092702 |
| A2ML1 | 0.74909 | 5.865669 | 3.698933 | 0.000245 | 0.001662 | 0.082216 |
| FGFBP2 | -0.6854 | 1.791965 | -3.6708 | 0.000273 | 0.001824 | -0.01622 |
| CA2 | 0.621886 | 7.171036 | 3.658151 | 0.000286 | 0.001897 | -0.06026 |
| RSAD2 | 0.636004 | 5.485659 | 3.648632 | 0.000297 | 0.001951 | -0.0933 |
| KRT75 | 0.844048 | 5.620365 | 3.639043 | 0.000308 | 0.002014 | -0.12651 |
| AKR1C3 | -0.71431 | 5.606238 | -3.61347 | 0.000339 | 0.002189 | -0.21465 |
| RNASE7 | 0.622432 | 5.377902 | 3.609496 | 0.000344 | 0.002212 | -0.2283 |
| CARD18 | 0.645362 | 2.867653 | 3.608179 | 0.000346 | 0.002219 | -0.23282 |
| CLDN17 | 0.637314 | 2.166002 | 3.587628 | 0.000373 | 0.00237 | -0.30314 |
| CHI3L1 | 0.641773 | 5.126774 | 3.584678 | 0.000377 | 0.00239 | -0.31321 |
| MMP1 | 0.763756 | 9.898838 | 3.579362 | 0.000385 | 0.00243 | -0.33132 |
| CRYAB | 0.607952 | 6.622741 | 3.575112 | 0.000391 | 0.002465 | -0.34579 |
| LY6G6C | 0.737262 | 5.035219 | 3.566665 | 0.000403 | 0.002528 | -0.37449 |
| RPTN | 0.8307 | 3.163756 | 3.532333 | 0.000458 | 0.002796 | -0.49047 |
| NTS | -0.96376 | 2.262313 | -3.51413 | 0.000489 | 0.002954 | -0.55156 |
| CEACAM6 | 0.720454 | 6.156001 | 3.511068 | 0.000495 | 0.00298 | -0.56179 |
| IGFL1 | 0.856695 | 6.037189 | 3.473122 | 0.000568 | 0.003352 | -0.68803 |
| CXCL14 | 0.691887 | 9.199748 | 3.459389 | 0.000597 | 0.003483 | -0.73341 |
| IGHM | 0.802808 | 5.772565 | 3.456439 | 0.000603 | 0.003516 | -0.74313 |
| KRT23 | 0.668363 | 4.944657 | 3.427168 | 0.00067 | 0.003836 | -0.83919 |
| TMEM45B | 0.631845 | 4.523207 | 3.408228 | 0.000717 | 0.004072 | -0.90094 |
| TMPRSS11D | 0.739684 | 6.117588 | 3.400757 | 0.000736 | 0.004157 | -0.92521 |
| IGKC | 0.800212 | 8.253609 | 3.335416 | 0.000928 | 0.00501 | -1.1353 |
| CASP14 | 0.797205 | 3.976659 | 3.313378 | 0.001002 | 0.00533 | -1.20529 |
| CDKN2B | 0.696082 | 5.127261 | 3.306467 | 0.001026 | 0.005434 | -1.22714 |
| CLCA4 | 0.649251 | 3.607578 | 3.297372 | 0.001059 | 0.00557 | -1.25584 |
| GSTA1 | -0.67889 | 2.014611 | -3.29574 | 0.001065 | 0.005581 | -1.261 |
| CXCL10 | 0.816083 | 7.441008 | 3.286265 | 0.001101 | 0.005742 | -1.29079 |
| KLK10 | 0.656846 | 7.529895 | 3.280073 | 0.001124 | 0.005844 | -1.31022 |
| MAL | 0.90131 | 4.505275 | 3.274639 | 0.001146 | 0.005944 | -1.32725 |
| SPINK6 | 0.869954 | 3.985023 | 3.250715 | 0.001244 | 0.006348 | -1.40189 |
| SYNPO2 | 0.598072 | 3.268381 | 3.246649 | 0.001261 | 0.00642 | -1.41452 |
| IGLL5 | 0.725433 | 5.227667 | 3.241629 | 0.001283 | 0.006503 | -1.4301 |
| CALB1 | -0.62582 | 1.89284 | -3.19718 | 0.001493 | 0.007359 | -1.56704 |
| KLK5 | 0.827514 | 7.045052 | 3.149149 | 0.001755 | 0.008375 | -1.71297 |
| SPRR2C | 0.826278 | 6.128543 | 3.143834 | 0.001786 | 0.008483 | -1.72899 |
| CEACAM5 | 0.653755 | 4.340854 | 3.02978 | 0.002599 | 0.011474 | -2.06654 |
| KLK12 | 0.685492 | 4.870771 | 2.962528 | 0.003225 | 0.013595 | -2.25998 |
| PLA2G2A | 0.639847 | 3.163065 | 2.935455 | 0.003514 | 0.014562 | -2.33668 |
| LCN2 | 0.625122 | 8.112914 | 2.925759 | 0.003623 | 0.01495 | -2.36399 |
| SCARNA15 | -0.78875 | 1.521113 | -2.92395 | 0.003644 | 0.015015 | -2.36908 |
| PRR9 | 0.749653 | 3.912258 | 2.912121 | 0.003781 | 0.015489 | -2.40225 |
| MB | 0.82879 | 4.418687 | 2.908177 | 0.003829 | 0.015627 | -2.41328 |
| MUCL1 | 0.640005 | 3.054713 | 2.893612 | 0.004007 | 0.01622 | -2.4539 |
| PMS2P8 | -0.69274 | 1.359391 | -2.87081 | 0.004302 | 0.017219 | -2.51709 |
| IGLC1 | -0.98189 | 1.914756 | -2.86582 | 0.004369 | 0.017437 | -2.53086 |
| AC091133.1 | -0.76004 | 1.497295 | -2.86207 | 0.00442 | 0.017599 | -2.54118 |
| IDO1 | 0.602496 | 4.513594 | 2.859862 | 0.00445 | 0.017688 | -2.54726 |
| XBP1 | -0.9008 | 1.780982 | -2.85102 | 0.004573 | 0.018055 | -2.57154 |
| SNORA21 | -0.62448 | 1.231075 | -2.8357 | 0.004794 | 0.018771 | -2.61344 |
| CRNN | 0.959801 | 4.464365 | 2.76183 | 0.006 | 0.022424 | -2.81248 |
| IGLV6-57 | 0.622217 | 4.73436 | 2.713783 | 0.006926 | 0.02512 | -2.93922 |
| NRAP | 0.612574 | 2.339293 | 2.651536 | 0.008316 | 0.029225 | -3.10022 |
| KLK14 | 0.613917 | 3.806676 | 2.592845 | 0.009851 | 0.033451 | -3.24871 |
| UPK1B | -0.6138 | 2.323014 | -2.51061 | 0.012428 | 0.040185 | -3.45135 |
| MYH2 | 0.804976 | 3.425166 | 2.474546 | 0.013735 | 0.043432 | -3.53823 |
| CXCL11 | 0.618792 | 4.994611 | 2.455079 | 0.01449 | 0.045324 | -3.58462 |
| MYBPC2 | 0.607012 | 2.937011 | 2.421614 | 0.015875 | 0.048722 | -3.66353 |

**Table S2**. Significant genes generated in Univariate Cox regression analysis

| Genes | HR | HR.95L | HR.95H | pvalue |
| --- | --- | --- | --- | --- |
| AOC1 | 0.800177 | 0.696715 | 0.919004 | 0.001601 |
| CD1A | 0.889956 | 0.801556 | 0.988106 | 0.028952 |
| CCL22 | 0.780119 | 0.687542 | 0.885162 | 0.000117 |
| FCER1A | 0.883893 | 0.793503 | 0.984581 | 0.024943 |
| CD1E | 0.849314 | 0.727684 | 0.991274 | 0.038350 |
| S100B | 0.863698 | 0.759719 | 0.981909 | 0.025161 |
| NUF2 | 1.204274 | 1.038468 | 1.396553 | 0.013917 |
| CKS2 | 1.359423 | 1.159650 | 1.593611 | 0.000153 |
| BLNK | 0.889210 | 0.795186 | 0.994351 | 0.039464 |
| KAT2B | 0.833257 | 0.711577 | 0.975743 | 0.023522 |
| UBE2T | 1.251252 | 1.073932 | 1.457851 | 0.004043 |
| CD1C | 0.842758 | 0.728112 | 0.975456 | 0.021845 |
| MND1 | 1.233226 | 1.048886 | 1.449963 | 0.011157 |
| CSF2RB | 0.812051 | 0.720758 | 0.914909 | 0.000623 |
| PLA2G2F | 0.861703 | 0.757076 | 0.980791 | 0.024219 |
| SDR9C7 | 0.908374 | 0.848372 | 0.972620 | 0.005848 |
| AURKB | 1.239245 | 1.060575 | 1.448016 | 0.006927 |
| PBK | 1.156074 | 1.007796 | 1.326168 | 0.038373 |
| CCR7 | 0.829841 | 0.744166 | 0.925380 | 0.000794 |
| RFC4 | 1.249883 | 1.077650 | 1.449642 | 0.003193 |
| FCGBP | 0.873800 | 0.796702 | 0.958358 | 0.004203 |
| SLC24A3 | 0.889247 | 0.799348 | 0.989256 | 0.030880 |
| BPIFC | 0.885359 | 0.807204 | 0.971082 | 0.009814 |
| LYNX1 | 0.866679 | 0.798934 | 0.940168 | 0.000570 |
| CLEC10A | 0.841741 | 0.741900 | 0.955019 | 0.007486 |
| CD5 | 0.792808 | 0.689642 | 0.911406 | 0.001098 |
| TMEM86A | 0.848732 | 0.728714 | 0.988516 | 0.034993 |
| NAPSB | 0.858565 | 0.762996 | 0.966106 | 0.011320 |
| P2RY13 | 0.868262 | 0.754465 | 0.999223 | 0.048746 |
| TMPRSS13 | 0.838728 | 0.745805 | 0.943229 | 0.003330 |
| S100A7A | 0.928596 | 0.876928 | 0.983308 | 0.011205 |
| RIMS3 | 0.777118 | 0.675710 | 0.893745 | 0.000408 |
| C10orf99 | 0.940366 | 0.887562 | 0.996311 | 0.037041 |
| LTB | 0.819335 | 0.719277 | 0.933312 | 0.002713 |
| TGM5 | 0.866703 | 0.780785 | 0.962075 | 0.007235 |
| RDH12 | 0.926487 | 0.863042 | 0.994596 | 0.034885 |
| TMEM79 | 0.889624 | 0.792899 | 0.998149 | 0.046425 |
| SPINK5 | 0.896727 | 0.843368 | 0.953462 | 0.000497 |
| RASSF2 | 0.831336 | 0.728668 | 0.948470 | 0.006021 |
| RASGRP1 | 0.881297 | 0.784727 | 0.989749 | 0.032846 |
| SLURP1 | 0.943210 | 0.899953 | 0.988545 | 0.014649 |
| CST7 | 0.864173 | 0.768313 | 0.971994 | 0.014954 |
| ANKRD22 | 0.871497 | 0.782544 | 0.970560 | 0.012281 |
| TRAC | 0.861243 | 0.779078 | 0.952073 | 0.003500 |
| CDSN | 0.920448 | 0.856422 | 0.989259 | 0.024226 |
| CXCR3 | 0.866418 | 0.763214 | 0.983577 | 0.026701 |
| PDZK1IP1 | 0.918033 | 0.849859 | 0.991675 | 0.029833 |
| SLCO2A1 | 0.886482 | 0.793061 | 0.990907 | 0.033944 |
| LCE3D | 0.925628 | 0.882990 | 0.970326 | 0.001319 |
| CD2 | 0.884551 | 0.805469 | 0.971398 | 0.010250 |
| BNIPL | 0.878842 | 0.799766 | 0.965736 | 0.007259 |
| KRT80 | 0.890680 | 0.818412 | 0.969330 | 0.007330 |
| WFDC12 | 0.925481 | 0.877821 | 0.975729 | 0.004095 |
| IVL | 0.922012 | 0.865846 | 0.981822 | 0.011339 |
| APCDD1 | 0.836843 | 0.739834 | 0.946573 | 0.004606 |
| IL2RG | 0.837935 | 0.750431 | 0.935643 | 0.001677 |
| SPNS2 | 0.843586 | 0.754112 | 0.943676 | 0.002946 |
| CD3E | 0.884410 | 0.792609 | 0.986843 | 0.028033 |
| CD27 | 0.852039 | 0.767468 | 0.945930 | 0.002681 |
| PGLYRP4 | 0.884612 | 0.813180 | 0.962319 | 0.004316 |
| VSIG10L | 0.891396 | 0.822618 | 0.965924 | 0.005013 |
| MUC15 | 0.886529 | 0.814856 | 0.964505 | 0.005107 |
| SERPINB7 | 0.900232 | 0.833193 | 0.972666 | 0.007771 |
| CCR5 | 0.886539 | 0.792403 | 0.991858 | 0.035491 |
| FMOD | 0.893570 | 0.801005 | 0.996831 | 0.043712 |
| ACER1 | 0.864916 | 0.763299 | 0.980061 | 0.022857 |
| S100A7 | 0.934925 | 0.890127 | 0.981977 | 0.007232 |
| SLC6A14 | 0.926160 | 0.861029 | 0.996218 | 0.039224 |
| CNFN | 0.894410 | 0.847231 | 0.944217 | 0.000054 |
| KRT8 | 1.087675 | 1.019760 | 1.160113 | 0.010625 |
| LYPD3 | 0.894222 | 0.812253 | 0.984462 | 0.022654 |
| KRT18 | 1.202411 | 1.075846 | 1.343865 | 0.001161 |
| FOXN1 | 0.866388 | 0.786922 | 0.953879 | 0.003478 |
| ENDOU | 0.882321 | 0.812099 | 0.958615 | 0.003088 |
| TPSAB1 | 0.829120 | 0.746895 | 0.920397 | 0.000437 |
| POU3F1 | 0.883963 | 0.795575 | 0.982171 | 0.021753 |
| CALML5 | 0.924262 | 0.877020 | 0.974049 | 0.003259 |
| SBSN | 0.932215 | 0.885607 | 0.981276 | 0.007313 |
| S100A9 | 0.912518 | 0.845292 | 0.985091 | 0.019043 |
| LCE2B | 0.899224 | 0.829109 | 0.975267 | 0.010329 |
| SPRR2G | 0.943839 | 0.903693 | 0.985769 | 0.009153 |
| KLK6 | 0.934825 | 0.884485 | 0.988029 | 0.017015 |
| SLC5A1 | 0.887833 | 0.807003 | 0.976759 | 0.014574 |
| IGHG1 | 0.924313 | 0.863993 | 0.988845 | 0.022268 |
| ALOX12B | 0.923159 | 0.868675 | 0.981060 | 0.009994 |
| SCEL | 0.915208 | 0.851649 | 0.983512 | 0.015835 |
| CYSRT1 | 0.922436 | 0.860347 | 0.989007 | 0.023155 |
| CPA3 | 0.880642 | 0.804005 | 0.964585 | 0.006216 |
| SPRR2B | 0.920479 | 0.878263 | 0.964724 | 0.000542 |
| FST | 1.109071 | 1.011981 | 1.215476 | 0.026776 |
| KRT78 | 0.900803 | 0.844606 | 0.960739 | 0.001480 |
| ASPRV1 | 0.927121 | 0.875125 | 0.982207 | 0.010181 |
| SPINK7 | 0.907114 | 0.861656 | 0.954970 | 0.000202 |
| SLPI | 0.911741 | 0.843401 | 0.985618 | 0.020105 |
| CRCT1 | 0.918270 | 0.871987 | 0.967009 | 0.001232 |
| PRSS27 | 0.896008 | 0.826314 | 0.971581 | 0.007865 |
| PI3 | 0.934056 | 0.886902 | 0.983717 | 0.009849 |
| TPSB2 | 0.909377 | 0.831559 | 0.994476 | 0.037404 |
| SPRR1B | 0.940748 | 0.888214 | 0.996389 | 0.037220 |
| PLA2G3 | 0.907599 | 0.833183 | 0.988661 | 0.026335 |
| KLK11 | 0.922036 | 0.858360 | 0.990436 | 0.026204 |
| IL36RN | 0.902404 | 0.845983 | 0.962588 | 0.001824 |
| CAPNS2 | 0.916420 | 0.847962 | 0.990404 | 0.027568 |
| CCL21 | 0.919171 | 0.858485 | 0.984146 | 0.015582 |
| POF1B | 0.912826 | 0.847133 | 0.983614 | 0.016687 |
| CD79A | 0.891383 | 0.819946 | 0.969042 | 0.006980 |
| S100A12 | 0.925079 | 0.869634 | 0.984059 | 0.013528 |
| RPL39L | 1.097545 | 1.011829 | 1.190522 | 0.024870 |
| SPRR1A | 0.934163 | 0.888097 | 0.982619 | 0.008302 |
| A2ML1 | 0.920722 | 0.862822 | 0.982507 | 0.012684 |
| AKR1C3 | 1.069353 | 1.002126 | 1.141091 | 0.042964 |
| CLDN17 | 0.917310 | 0.846219 | 0.994374 | 0.035990 |
| LY6G6C | 0.932675 | 0.874792 | 0.994389 | 0.032999 |
| RPTN | 0.920292 | 0.867524 | 0.976269 | 0.005830 |
| TMPRSS11D | 0.912919 | 0.859201 | 0.969995 | 0.003234 |
| IGKC | 0.943581 | 0.890892 | 0.999386 | 0.047601 |
| CLCA4 | 0.916861 | 0.851210 | 0.987576 | 0.022035 |
| KLK10 | 0.924106 | 0.864319 | 0.988028 | 0.020727 |
| SPINK6 | 0.912628 | 0.864746 | 0.963162 | 0.000884 |
| IGLL5 | 0.938751 | 0.883157 | 0.997845 | 0.042436 |
| CEACAM5 | 0.939688 | 0.883221 | 0.999765 | 0.049138 |
| KLK12 | 0.901188 | 0.848828 | 0.956777 | 0.000657 |
| CRNN | 0.951629 | 0.913404 | 0.991453 | 0.017771 |
| IGLV6-57 | 0.936521 | 0.880214 | 0.996431 | 0.038173 |
| KLK14 | 0.936301 | 0.881005 | 0.995067 | 0.034076 |

**Table S3.** Primers used in this study.

| Primers | Sequences | |
| --- | --- | --- |
| GAPDH-F | 5’-GTCTCCTCTGACTTCAACAGCG-3’ | |
| GAPDH-R | 5’-ACCACCCTGTTGCTGTAGCCAA-3’ | |
| CKS2-F | 5’-GCGCTCTCGTTTCATTTT-3’ |  |
| CKS2-R | 5’-CCAAGTCTCCTCCACTCCT-3’ |  |
| RIMS3-F | 5’-GGCTACAGGGAATCAAAGG-3’ |  |
| RIMS3-R | 5’-AGGGAATCATGGGGAGAG-3’ |  |
| TRAC-F | 5’-TGGCCTAACCCTGATCCTCTT-3’ |  |
| TRAC-R | 5’-GGATTTAGAGTCTCTCAGCTGGTACAC-3’ |  |
| FMOD-F | 5’-AACCAGATCACCTCCATCC-3’ |  |
| FMOD-R | 5’-TCCTGCCCACCTTATCAC-3’ |  |
| CALML5-F | 5’-GGTCGGTGGATGGGAAAC-3’ |  |
| CALML5-R | 5’-CCCAAGGTCTGGAGGCA-3’ |  |
| SPINK7-F | 5’-TTCATCATCCCAGGCTCT-3’ |  |
| SPINK7-R | 5’-GGGTTTGTAGGGGTAGCAC-3’ |  |
